# Src inhibition potentiates MCL-1 antagonist activity in acute myeloid leukemia

**DOI:** 10.1038/s41392-025-02125-x

**Published:** 2025-02-10

**Authors:** Xiaoyan Hu, Lin Li, Jewel Nkwocha, Maciej Kmieciak, Shengzhe Shang, L. Ashley Cowart, Yang Yue, Katsuhisa Horimoto, Adam Hawkridge, Arjun Rijal, Adolfo G. Mauro, Fadi N. Salloum, Lori Hazlehurst, Konstantinos Sdrimas, Zackary Moore, Liang Zhou, Gordon D. Ginder, Steven Grant

**Affiliations:** 1 Division of Hematology/Oncology, Department of Internal Medicine, Virginia Commonwealth University, Richmond, VA USA; 2 Massey Cancer Center, Virginia Commonwealth University, Richmond, VA USA; 3 Department of Biochemistry and Molecular Biology, Virginia Commonwealth University, Richmond, VA USA; 4 Office of the Vice President for Research Infrastructure, Virginia Commonwealth University, Richmond, VA USA; 5 SOCIUM Inc., Tokyo, 1350064 Japan; 6 Department of Pharmaceutics, Virginia Commonwealth University, Richmond, VA USA; 7 Pauley Heart Center, Division of Cardiology, Department of Internal Medicine, Virginia Commonwealth University, Richmond, VA USA; 8 Department of Pharmaceutical Science, WVU Cancer Institute, Morgantown, WV USA; 9 Department of Medical Oncology, WVU Cancer Institute, Morgantown, WV USA; 10 Department of Translational Medicine, Asklepios BioPharmaceutical, Inc., Durham, NC USA

**Keywords:** Haematological cancer, Haematological cancer, Translational research

## Abstract

The importance of MCL-1 in leukemogenesis has prompted development of MCL-1 antagonists e.g., S63845, MIK665. However, their effectiveness in acute myeloid leukemia (AML) is limited by compensatory MCL-1 accumulation via the ubiquitin proteasome system. Here, we investigated mechanisms by which kinase inhibitors with Src inhibitory activity e.g., bosutinib (SKI-606) might circumvent this phenomenon. MCL-1 antagonist/SKI-606 co-administration synergistically induced apoptosis in diverse AML cell lines. Consistently, Src or MCL-1 knockdown with shRNA markedly sensitized cells to MCL-1 inhibitors or SKI-606 respectively, while ectopic MCL-1 expression significantly diminished apoptosis. Mechanistically, MCL-1 antagonist exposure induced MCL-1 up-regulation, an event blocked by Src inhibitors or Src shRNA knock-down. MCL-1 down-regulation was associated with diminished transcription and increased K48-linked degradative ubiquitination. Enhanced cell death depended functionally upon down-regulation of phosphorylated STAT3 (Tyr705/Ser727) and cytoprotective downstream targets c-Myc and BCL-xL, as well as BAX/BAK activation, and NOXA induction. Importantly, the Src/MCL-1 inhibitor regimen robustly killed primary AML cells, including primitive progenitors, but spared normal hematopoietic CD34^+^ cells and human cardiomyocytes. Notably, the regimen significantly improved survival in an MV4-11 cell xenograft model, while reducing tumor burden in two patient-derived xenograft (PDX) AML models and increased survival in a third. These findings argue that Src inhibitors such as SKI-606 potentiate MCL-1 antagonist anti-leukemic activity in vitro and in vivo by blocking MCL-1 antagonist-mediated cytoprotective MCL-1 accumulation by promoting degradative ubiquitination, disrupting STAT-3-mediated transcription, and inducing NOXA-mediated MCL-1 degradation. They also suggest that this strategy may improve MCL-1 antagonist efficacy in AML and potentially other malignancies.

## Introduction

Acute myeloid leukemia (AML) is a malignancy that stems from the failure of primitive myeloid progenitors or hematopoietic stem cells to undergo an orderly process of differentiation.^1^ It represents approximately 25% of leukemias in adults and is therefore the most common form of leukemia in this population.^2^ However, despite the introduction of novel agents targeting specific genetic aberrations (e.g., FLT3, IDH1/2, NPM1 etc.),^3^ as well as the curative potential of bone marrow transplantation in a subset of patients,^4^ acute myeloid leukemia (AML) remains incurable in most patients, particularly those with relapsed/refractory disease.^5^ Consequently, more effective approaches to AML treatment are urgently needed.

Like many malignancies, AML is associated with deregulation of pro- and anti-apoptotic regulatory proteins.^6^ These consist of pro-survival proteins (e.g., BCL2, BCL-xL, MCL-1, A1) and their pro-apoptotic counterparts e.g., BAX, BAK, BAD, PUMA, and NOXA, among others.^7^ Perturbations in anti-apoptotic proteins in AML e.g., BCL2 have prompted development of BH3-mimetics such as the specific BCL2 antagonist venetoclax,^8^ approved for older AML patients when combined with the hypomethylating agent 5-azacytidine.^9^ However, a major mechanism of venetoclax resistance involves up-regulation of the anti-apoptotic protein MCL-1 which is weakly bound to this agent.^10^ MCL-1 is a short-lived protein (T1/2 ~ 2–4 h),^11^ frequently up-regulated in AML, and is implicated in AML stem cell-like cell survival.^12^ MCL-1 is validated as a target in other hematologic malignancies e.g., multiple myeloma^13^ and non-Hodgkins lymphoma,^14^ as well as diverse solid tumor malignancies, including lung cancer,^15^ breast cancer,^16^ and colon cancer,^17^ among others.^18^ These considerations prompted the development of MCL-1 antagonists e.g., S63845, MIK665 that have entered the clinical arena (Clin Trial.Gov; NCT04629443). Furthermore, pre-clinical studies show that combining venetoclax with MCL-1 antagonists induces AML cell apoptosis,^19^ supporting the clinical development of this concept.^19^

Src family kinases (SFKs) are implicated in leukemogenesis^20^ and activation correlates with poor prognosis.^21^ Moreover, tyrosine kinase inhibitors such as dasatinib and bosutinib exhibiting SFK-inhibitory actions show pre-clinical activity in AML models,^22^ and are approved in chronic myeloid leukemia (CML).^23^ Notably, there is evidence in AML that Src inhibition acts by diminishing MCL-1 abundance.^24^ For example, in multiple myeloma cells, SFKs have been implicated in MCL-1 regulation through a process involving the phosphatase of regenerating liver-3 (PRL-3), and Src inhibitors down-regulate this protein.^25^ In Philadelphia^+^ acute lymphocytic leukemia (ALL) cells, tyrosine kinase inhibitors (e.g., dasatinib, bosutinib) with SFK inhibitory activity^26^ diminish MCL-1 expression and induce apoptosis.^27^ We have reported that Src inhibitors down-regulate MCL-1 in myeloid leukemia cells.^28^

Several studies have demonstrated that B-cell lymphoma 2 (Bcl-2) Homology 3 (BH3)-mimetics induce MCL-1 up-regulation as a compensatory response in leukemia cells. For example, venetoclax induces MCL-1 up-regulation in AML cells,^29^ and recently MCL-1 antagonists have been shown to potentiate MCL-1 accumulation in chronic lymphocytic leukemia (CLL) cells by diminishing MCL-1 ubiquitination and degradation.^30^ Interestingly, MCL-1 inhibitors enhance the ability of Src antagonists to inhibit breast cancer cell metastasis.^31^ Together, these studies provide a rationale for employing Src inhibitors to reverse MCL-1 accumulation in AML cells induced by MCL-1 inhibitors. The goal of this study was to validate this hypothesis and identify mechanisms by which clinically relevant Src inhibitors prevent MCL-1 accumulation by MCL-1 antagonists, and enhance anti-AML activity.

## Results

### Interactions between MCL-1 and Src inhibitors in AML cells

**Fig. 1:**
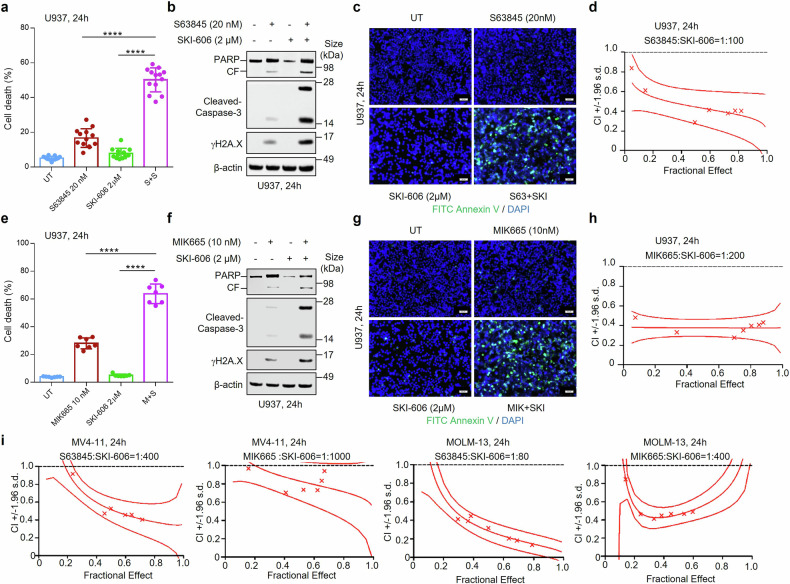
MCL-1 inhibitors interact synergistically with SKI-606 to induce apoptosis in AML cells. **a** U937 cells were exposed to the indicated concentrations of S63845 ± SKI-606 for 24 h, followed by flow cytometric analysis of cell death after staining with 7-AAD (*n* = 13 in each group). Values represent the mean % ± standard deviations (SD). **b** Cells were incubated with S63845 ± SKI-606 for 24 h, after which PARP, cleaved-Caspase-3 and γH2A.X were monitored by immunoblotting analysis. β-actin was used as a loading control to ensure equal protein loading and transfer. **c** Cells were exposed (24 h) to S63845 ± SKI-606, followed by Annexin V and DAPI staining using fluorescence microscopy. Scale bar = 50 µm. **d** Cells were exposed (24 h) to varying concentrations of S63845 ± SKI-606 at a fixed ratio (1:100), after which the percentage of 7-AAD^+^ cells was determined. Median dose-effect analysis was then employed to characterize the nature of the interaction between these agents. Combination index values < 1.0 denote a synergistic interaction. **e**–**h** U937 cells were exposed to the indicated concentrations of MIK665 ± SKI-606 for 24 h. Assays were performed as in (**a**–**d**). **e** *n* = 7 in each group. **i** Synergist**i**c interaction between S63845 or MIK665 and SKI-606 were tested in two additional AML cell lines. CF, cleavage fragment; DAPI, 4′,6-diamidino-2-phenylindole. ****P < 0.0001

We first sought to determine whether the anti-leukemic activity of clinically relevant MCL-1 antagonists could be enhanced by Src inhibitors. Exposure (24 h) to the MCL-1 antagonist S63845 (20 nM) or SKI-606 (2 μM) individually minimally affected U937 cell viability, but together dramatically increased cell death, manifested by PARP and caspase-3 cleavage and γH2A.X formation (Fig. 1a, b, Supplementary Fig. S1a). Annexin V staining demonstrated a marked increase in apoptosis (Fig. 1c), which was synergistic over a range of concentrations by Median Dose Effect analysis (Fig. 1d). Identical results were obtained with the MCL-1 antagonist MIK665 (Fig. 1e–h, Supplementary Fig. S1b). Similar synergistic interactions occurred in multiple AML cell lines e.g., MV4-11, MOLM-13 and OCI-AML3 exposed to S63845 + SKI-606 or MIK665 + SKI-606 (Fig. 1i, Supplementary Fig. S1c–j).

To extend findings to another Src inhibitor, parallel studies were performed in MV4-11 cells exposed to the tyrosine kinase/Src inhibitor dasatinib. Coadministration (24 h) of dasatinib (2 μM) with S63845 (2 nM) or MIK665 (1 nM) synergistically increased apoptosis (Supplementary Fig. S2a–c). Finally, co-administration of dasatinib and S63845 or MIK665 sharply increased cell death in U937 cells (Supplementary Fig. S2d), indicating that Src inhibitors synergistically increase MCL-1 inhibitor anti-leukemic activity.

### Genetic validation of MCL-1 and Src actions in AML cells

To confirm on-target effects of MCL-1 inhibitors, U937 cells expressing MCL-1 shRNA constructs (clone #4 and #5) were generated. These cells displayed diminished MCL-1 expression (Supplementary Fig. S3a, inset), and were significantly more sensitive to SKI-606 than empty-vector controls (Supplementary Fig. S3a), exhibiting increased PARP and caspase-3 cleavage as well as γH2A.X generation (Supplementary Fig. S3b). Parallel studies in U937 cells in which Src was knocked down with shRNA (clones #1 and #5; Supplementary Fig. S3c, inset) revealed that these cells were significantly more sensitive to 20 nM S63845-induced cell death than controls (Supplementary Fig. S3c; left panel), and displayed increased PARP and caspase-3 cleavage as well as γH2A.X formation (Supplementary Fig. S3d; left panel). Compatible events occurred in cells exposed to 10 nM MIK665 (Supplementary Fig. S3c; right panel and Supplementary Fig. S3d, right panel). Finally, consistent findings occurred in MV4-11 cells e.g., significantly increased lethality following SKI-606 exposure in MCL-1 knock-down cells (Supplementary Fig. S4a, b) and increased S63845- and MIK665-induced cell death in Src knock-down cells (Supplementary Fig. S4c, d). These findings confirm the on-target actions of Src and MCL-1 antagonists in promoting AML cell death.

### MCL-1 down-regulation plays a functional role in regimen-mediated cell death

**Fig. 2:**
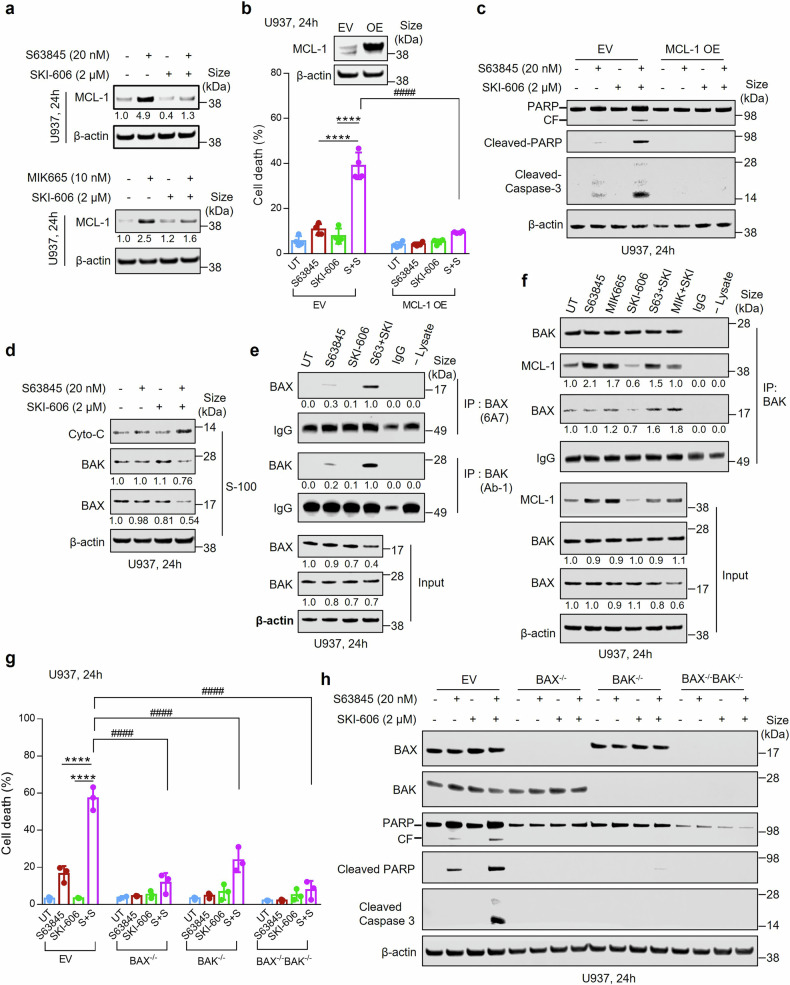
MCL-1 down-regulation and activation of BAX and BAK are required for S63845/SKI-606-mediated apoptosis. **a** U937 cells were exposed to the indicated concentrations of S63845 or MIK665 ± SKI-606 for 24 h, after which the level of MCL-1 was determined by western blot analysis. Numerals under the blots correspond to densitometric readings normalized to untreated controls (1.0). **b**, **c** Ectopic expression of MCL-1 in U937 cells. **b** Cells were treated (24 h) with 20 nM S63845 ± 2 µM SKI-606 and cell death was determined by 7-AAD staining and flow cytometric analysis (*n* = 4 in each group). Inset, levels of MCL-1 by western blot after overexpression. **c** Western blot analysis of PARP, cleaved- PARP, as well as cleaved-Caspase-3 was performed. β-actin was assayed to ensure equivalent loading and transfer. **d** U937 cells were exposed to S63845 (20 nM) and SKI-606 (2 μM) alone or in combination for 24 h, after which subcellular fractions were obtained and subjected to western blot analysis to monitor the release of cytochrome **c**, BAK, and BAX into the cytosol. S-100, cytosol; Cyto c = cytochrome c. Numerals under the blots correspond to densitometric readings normalized to untreated controls (1.0). **e** U937 cells were exposed to S63845 ± SKI-606 for 24 h, after which cells were lysed in buffer containing 1% CHAPS; conformationally changed BAX and BAK proteins were immunoprecipitated using anti-BAX 6A7 and anti-BAK Ab1 antibodies, respectively, and subjected to western blot analysis using polyclonal BAX or BAK antibodies. **f** Following 24 h treatment, U937 cells were lysed in buffer and immunoprecipitated (IP) using anti-BAK antibody, followed by western blot analysis using anti-BAK, anti-BAX, or anti-MCL-1 antibodies as indicated. For all IP assay, IPs without cell lysate (-lysate) and/or with IgG (instead of primary antibodies) were carried out as controls; Input lysates were also subjected to western blot analysis to monitor relative protein levels. IgG levels are shown to ensure equal loading of IP antibodies. **g** BAK and/or BAX CRISPR knockout U937 cells were incubated with S63845 ± SKI-606 for 24 h. Cell death was determined using 7-AAD staining and flow cytometry. Values represent the mean % ± SD for three separate experiments. **h** BAK, BAX, PARP, cleaved-PARP and cleaved-Caspase-3 were detected by western blot. β-actin was assayed to ensure equivalent loading and transfer. CF, cleavage fragment; EV, empty vector; OE, overexpression. ****P < 0.0001; ^####^*P* < 0.0001

To validate further the role of MCL-1 in MCL-1/Src inhibitor interactions, western blot analysis was performed in U937 cells exposed to agents alone and in combination. As reported in CLL cells,^30^ S63845 sharply increased MCL-1 expression in AML cells. However, SKI-606 co-administration blocked this event (Fig. 2a, upper panel). Similar results occurred with MIK665 (Fig. 2a, lower panel) and in MV4-11, MOLM-13, and OCI-AML3 cells exposed to S63845 and SKI-606 (Supplementary Fig. S5a). Notably, cells ectopically expressing MCL-1 were protected from S63845/SKI-606-mediated cell death compared to empty-vector controls (Fig. 2b, c). Compatible results were obtained with MIK665 (Supplementary Fig. S5b, c). Similarly, MCL-1 ectopic expression in MV4-11 cells substantially blocked S63845/SKI-606 or MIK665/SKI-606-mediated cell death (Supplementary Fig. S6a, b), arguing that prevention of MCL-1 antagonist-mediated MCL-1 up-regulation contributes functionally to Src antagonist mediated potentiation of MCL-1 inhibitor-induced leukemic cell death.

### Combined MCL-1 inhibitor/SKI-606-mediated lethality requires BAK and BAX

To investigate roles of the multi-domain pro-apoptotic proteins BAX and BAK in these events, immunoprecipitation analysis was performed. Combined exposure of U937 cells to S63845 and SKI-606 sharply increased cytosolic (S-100) cytochrome C and reduced cytosolic BAX and BAK, presumably secondary to nuclear translocation (Fig. 2d). Similar events occurred with MIK665 (Supplementary Fig. S5d), accompanied by a marked increase in BAX and BAK conformational change/activation for S63845 (Fig. 2e) or MIK665 (Supplementary Fig. S5e) in combination with SKI-606. Immunoprecipitation analysis revealed that either S63845 or MIK665 co-administration with SKI-606 a) prevented increased MCL-1 binding to BAK induced by MCL-1 antagonists alone, and b) increased BAX co-precipitating with BAK, presumably reflecting heterodimerization (Fig. 2f). Notably, BAK and BAX CRISPR knock-out cells displayed a sharp reduction in S63845/SKI-606-mediated cell death, which was essentially complete in dual BAX/BAK knock-out cells (Fig. 2g). These findings were recapitulated following exposure to MIK665 and SKI-606 (Supplementary Fig. S5f), as confirmed by western blot analysis of PARP and caspase-3 cleavage (Fig. 2h and Supplementary Fig. S5g).

Analogous studies were performed in parental MV4-11 cells as well as their BAX/BAK KO counteraparts. Combined S63845 and SKI-606 exposure increased BAX/BAK conformational change (Supplementary Fig. S6c), diminished MCL-1/BAK co-immunoprecipitation, and increased BAX/BAK heterodimerization in MV4-11 (Supplementary Fig. S6d). Moreover, cell killing by SKI-606 + S63845 or MIK665 was significantly reduced by BAK or BAX knockout, particularly in dual BAX/BAK KO cells (Supplementary Fig. S6e, f), arguing that Src/MCL-1 inhibitor mediated cell death is critically dependent upon BAX and BAK activation.

### The MCL-1 inhibitor/SKI-606 regimen induces apoptosis in a STAT3-dependent manner

**Fig. 3:**
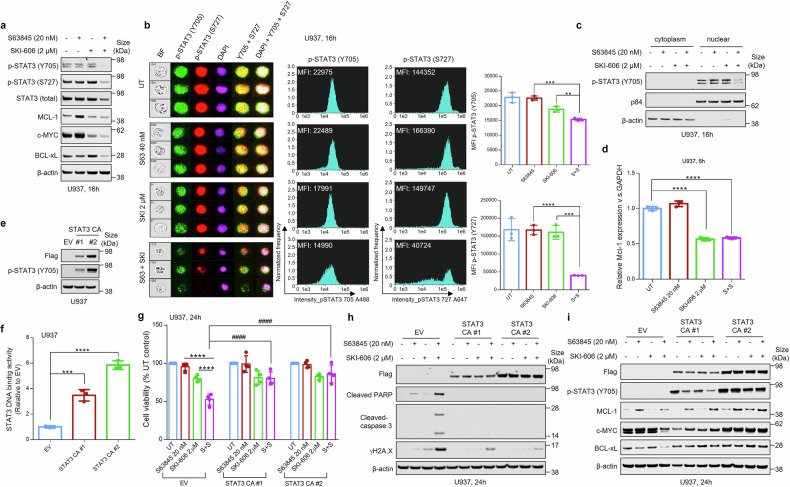
The S63845/SKI-606 regimen blocks phosphorylation of STAT3 (p-Y705/ p-S727) and diminishes MCL-1 transcription by reducing p-STAT3 (Y705) nuclear translocation. **a** Western blot analysis of p-STAT3 (Y705), p-STAT3 (S727), MCL-1, c-MYC, BCL-_X_L in U937 cells treated with 20 nM S63845 ± 2 µM SKI-606 for 16 h. **b** Cells were stained with p-STAT3 (Y705), p-STAT3 (Y727) and DAPI, and then visualized by fluorescence microscopy and by ImageStream analysis. Representative cells are shown (BF = brightfield). Histograms of p-STAT3 (Y705) and p-STAT3 (Y727) intensity and fold-change are shown. Representative data for at least three replicates. **c** U937 cells were treated with S63845 ± SKI-606 for 16 h. Nuclear and cytoplasmic extracts were collected for performance of p-STAT3 (Y705) immunoblotting assays. P84 and β-actin were used as loading controls for nuclear and cytoplasmic protein, respectively. **d** Relative mRNA expressions of MCL-1 was determined by real-time RT-PCR analysis (*n* = 3 in each group). GAPDH served as an internal control. **e**–**i** U937 cells were infected with a lentivirus harboring constitutively-active STAT3 (FLAG fusion), and STAT3-CA clone #1 and #2 were selected for the following experiments. **e** Western blot analysis was performed to test Flag and p-STAT3 (Y705) levels in STAT3-CA cells. **f** STAT3 DNA-binding ELISA was used to evaluate STAT3 activity in STAT3-CA cell (*n* = 3 in each group). **g** Cells were exposed (24 h) to indicated concentrations of S63845 ± SKI-606, followed by CellTiter-Glo® Luminescent Cell Viability Assay to monitor cell viability. Values represent the mean % ± SD for four separate experiments performed in triplicate. **h** Western blot analysis of FLAG, cleaved-PARP and cleaved-Caspase-3, as well as γH2A.X was performed. β-actin were assayed to ensure equivalent loading and transfer. **i** Western blot analysis of FLAG, p-STAT3 (Y705), MCL-1, C-MYC, as well as BCL-xL was performed. β-actin were assayed to ensure equivalent loading and transfer. **P < 0.01, ***P < 0.001, ****P < 0.0001; ^####^*P* < 0.0001

In light of evidence implicating SFKs in STAT3 activation,^32^ as well as the role of STAT3 in regulating MCL-1 transcription,^33^ we hypothesized that effects of Src inhibitors on MCL-1 antagonist-mediated anti-leukemic effects might involve STAT3. As shown in western blots (Fig. 3a), co-administration of S63845 and SKI-606 in U937 cells resulted in enhanced dephosphorylation of STAT3 at both Ser727 and Tyr705 activation sites while blocking MCL-1 up-regulation and down-regulating expression of the STAT3 targets c-MYC and BCL-xL. These events were confirmed by ImageStream analysis (Fig. 3b) and quantified (Fig. 3b, right panels). Combined exposure also diminished p-STAT3 Tyr705 nuclear translocation (Fig. 3c). Moreover, exposure of cells to SKI-606 ± S63845 significantly reduced MCL-1 transcription determined by qRT-PCR (Fig. 3d). Notably, results were similar for SKI given alone, arguing that Src inhibition is the primary driver underlying this phenomenon.

To assess the functional significance of these events, cells were infected with a lentivirus harboring constitutively-active STAT3 (FLAG fusion) (Fig. 3e), and exhibited significantly increased basal STAT3 DNA-binding activity (Fig. 3f). These cells were partially but significantly less sensitive to SKI-606/S63845-mediated reductions in viability (Fig. 3g) and exhibited diminished apoptosis compared to their empty-vector counterparts (Fig. 3h). Finally, cells expressing constitutively active STAT3 showed diminished MCL-1, c-MYC, and BCL-xL down-regulation following S63845/SKI-606 exposure than empty vector controls (Fig. 3i).

Similar findings occurred with MIK665 e.g., combined MIK665/SKI-606 exposure diminished p-STAT3 Tyr705 and Ser727, and down-regulated MCL-1, c-MYC, and BCL-xL (Supplementary Fig. S7a, b; quantified in right panels). Co-treatment also diminished p-STAT3-Tyr705 nuclear translocation (Supplementary Fig. S7c) and inhibited MCL-1 transcription (Supplementary Fig. S7d). Finally, cells expressing constitutively active STAT3 were significantly protected from the effects of MIK665/SKI-606, and exhibited less down-regulation of MCL-1, c-MYC, and BCL-xL following MIK665/SKI-606 treatment compared to controls (Supplementary Fig. S7e–g), as with S63845/SKI-606. Comparable results were also observed in MV4-11 cells (Supplementary Fig. S8a–f). Taken together, these findings argue that Src inhibitor-mediated STAT3 inactivation and resulting down-regulation of cytoprotective targets play significant functional roles in Src/MCL-1 antagonist-induced leukemic cell death.

### c-MYC and BCL-xL down-regulation play functional roles in MCL-1/SFK inhibitor lethality

To assess the functional significance of c-MYC and BCL-xL down-regulation, cells ectopically expressing these proteins were generated. U937 cells ectopically expressing c-MYC (Supplementary Fig. S9a) were significantly less sensitive to the SKI-606/S63845 and SKI-606/MIK665 regimens than their empty-vector controls (Supplementary Fig. S9b). Western blot analysis confirmed these results (Supplementary Fig. S9c). Similarly, cells ectopically expressing BCL-xL (Supplementary Fig. S9d) were significantly less sensitive to the SKI-606/MIK665 or SKI-606/S63845 regimens compared to controls (Supplementary Fig. S9e, f). These findings argue that the ability of Src disruption to inhibit STAT3 activation and diminish MCL-1, c-MYC, and BCL-xL expression contributes functionally to enhanced anti-leukemic activity.

### The MCL-1/SFK inhibitor regimen promotes NOXA and ubiquitination-related degradation of MCL-1

**Fig. 4:**
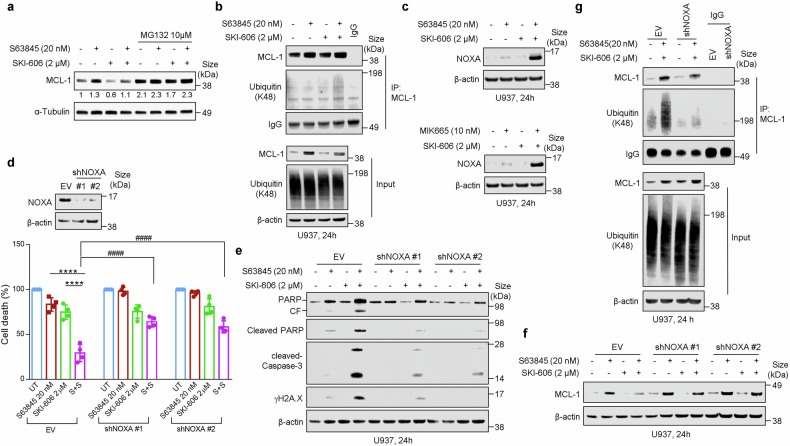
Co-treatment with S63845 and SKI-606 promotes the NOXA and ubiquitination-related degradation of MCL-1. **a** Effect of MG132 on MCL-1 expression in U937 cells treated with S63845 ± SKI-606. The cells were treated with 20 nM S63845 ± 2 µM SKI-606 for 16 h and then incubated with 10 µM MG132 for 2 h. **b** U937 cells were exposed to the indicated concentrations of S63845 ± SKI-606 for 24 h, after which cells were lysed in buffer and immunoprecipitated (IP) using anti-MCL-1 antibody, followed by western blot analysis using anti-Ubiquitin K48 antibody as indicated; Input lysates were also subjected to western blot analysis to monitor relative protein levels. IgG levels are shown to ensure equal loading of IP antibodies. **c** Western blot analysis showing expression of NOXA in U937 cells treated with S63845 or MIK665 ± SKI-606. **d**–**g** U937 cells were infected with a lentivirus harboring NOXA shRNA or EV, clones #1 and #2 were selected for the following experiments. **d** Cells were exposed to 20 nM S63845 ± 2 μM SKI-606 for 24 h, after which cell viability was determined by CellTiter-Glo® Luminescent Cell Viability Assay. Values represent the mean % ± SD for four separate experiments performed in triplicate. Inset: expression of NOXA by WB after infection with a lentivirus harboring EV or shNOXA. **e** Western blot analysis of PARP, cleaved-PARP, cleaved-Caspase-3, and γH2A.X was performed. β-actin controls were assayed to ensure equivalent loading and transfer. **f** Effect of NOXA depletion on MCL-1 expression. **g** Ubiquitination levels of MCL-1 in U937 NOXA knock-down cells treated with S63/SKI. IP with IgG (instead of primary antibodies) were carried out as controls. EV, empty vector, CF, cleavage fragment. ****P < 0.0001; ^####^*P* < 0.0001

Studies were then undertaken to elucidate mechanisms responsible for Src inhibitor-mediated abrogation of MCL-1 up-regulation by MCL-1 inhibitors. Co-exposure of U937 cells to S63845 and SKI-606 with the proteasome inhibitor MG-132 (10 µM) prevented MCL-1 down-regulation (Fig. 4a), implicating proteasomal degradation in this phenomenon. Combination treatment was associated with a marked increase in degradative K48 MCL-1 ubiquitination (Fig. 4b). In addition, co-treatment with SKI-606 and S63845 (or MIK655), triggered a sharp increase in NOXA expression (Fig. 4c), known to induce MCL-1 degradation.^34^

To evaluate the functional significance of NOXA induction, two shRNA knock-down clones were generated (Fig. 4d, inset). Each clone was significantly more resistant to S63845/SKI-606-mediated loss of viability than empty-vector controls (Fig. 4d), and displayed less PARP and caspase-3 cleavage (Fig. 4e). NOXA knockdown cells showed diminished MCL-1 down-regulation following S63845/SKI-606 exposure than controls (Fig. 4f), accompanied by a significant reduction in degradative K48-linked ubiquitination of MCL-1 compared to controls (Fig. 4g).

Identical findings were observed in U937 cells exposed to SKI-606 in combination with MIK665 (Supplementary Fig. S10a–f). In addition, MV4-11 cells exposed to SKI-606 + either S63845 or MIK665 exhibited marked K48 degradative ubiquitination and robust NOXA induction (Supplementary Fig. S11a and S11b). Collectively, these findings argue that Src inhibition blocks MCL-1 up-regulation and promotes leukemic cell death through multiple mechanisms e.g., STAT3 inactivation, proteasomal degradation, destructive K48 ubiquitination, and NOXA-mediated down-regulation.

### MCL-1/SFK inhibition sharply induces apoptosis in primary AML blasts in vitro but not in normal CD34^+^ cells

**Fig. 5:**
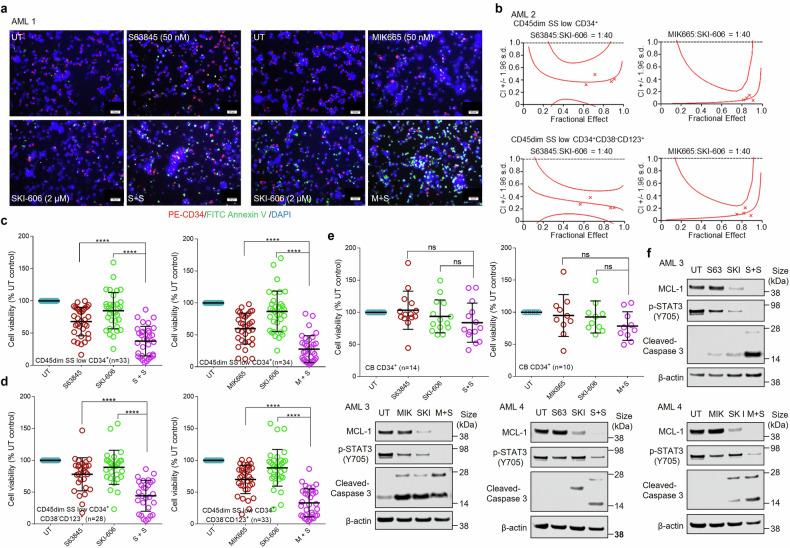
The MCL-1 inhibitor/SKI-606 regimen kills primary AML blasts but not normal CD34^+^ cells, blocks STAT3 phosphorylation (p-Y705), and down-regulates MCL-1. **a** Representative primary bone marrow cells from patients with AML were exposed to 50 nM S63845 or MIK665 ± 2 µM SKI-606 for 24 h, after which the cells were stained with PE-CD34, FITC-Annexin V and DAPI. Scale bar = 20 μm. **b** Primary AML patient samples were exposed (16–20 h) to varying concentrations of S63845 or MIK665 ± SKI-606 at a fixed ratio (1:40), after which the percentage of apoptotic (annexin V^+^) cells was determined. Median dose-effect analysis was then employed to characterize the nature of the interaction between these agents. Combination index values < 1.0 denote a synergistic interaction. **c** Assessment of cell viability in primary CD34^+^ AML cells following a 16–20 h exposure to 50 nM S63845 or MIK665 ± 2 µM SKI-606 by multi-color flow cytometric determination of Annexin V-FITC/7-AAD uptake. Lines indicate mean and SD. **d** Parallel experiments were conducted in primary AML specimens exhibiting a more primitive phenotype (CD34^+^CD123^+^CD38^-^). **e** Analogous experiments were carried out with primary cord blood (CB) CD34^+^ samples. **f** Western blot analysis of MCL-1, p- STAT3 (Y705) and cleavage of Caspase-3 in representative primary patient samples following exposure to S63845 or MIK665 50 nM ± SKI-606 2 µM for 16–20 h. β-actin controls were assayed to ensure equivalent loading and transfer. ****P < 0.0001; ns, not significant

Attempts were then made to extend these findings to primary AML blasts, clinical features of which are listed in Supplementary Table S2. Co-exposure (16–24 h) of CD34^+^ AML blasts to both S63845 + SKI-606 clearly increased green-staining annexin V-positive cells (Fig. 5a). Results were even more striking with MIK665 + SKI-606 (Fig. 5a). Median Dose Effect Analysis for both combinations in the CD45dim, side scatter low, CD34^+^ cell population yielded Combination Index (CI) values < 1.0, indicating synergistic interactions (Fig. 5b, upper panels). Similar results were obtained in a more primitive population,^35^ e.g., CD45 dim, side scatter low, CD34^+^, CD38^-^, CD123^+^ (Fig. 5b, lower panels). Analysis of cell death induced by SKI-606 + S63845 or MIK665 in bulk blast cell specimens (Fig. 5c) or more primitive cells (Fig. 5d) showed significant increases for combined versus individual exposure. In stark contrast, exposure of normal CD34^+^ cells to SKI-606 + S63845 (14 samples) or SKI-606 + MIK665 (10 samples) did not increase cell death (Fig. 5e). In view of the potential cardiotoxicity of BH3 mimetics,^36^ parallel studies utilizing a normal human adult ventricular cardiomyocyte cell line (AC16) showed that the combination regimen failed to increase cardiomyocyte cell death (Supplementary Fig. S12a, b). Western blot analysis was performed on bulk populations of blasts exposed to SKI-606 ± S63845 or MIK665 (Fig. 5f) and showed that combined treatment robustly induced STAT3 Tyr705 dephosphorylation, MCL-1 down-regulation, and caspase-3 cleavage. Each of these findings e.g., synergistic induction of apoptosis by SKI-606 + S63845 or MIK665, dephosphorylation of STAT3 Tyr705, down-regulation of MCL-1, and caspase-3 cleavage were recapitulated in multiple other primary bulk blast samples (Supplementary Fig. S13a–c).

### In vivo anti-leukemia efficacy of the MCL-1 inhibitor/SKI-606 regimen in mouse xenograft models

**Fig. 6:**
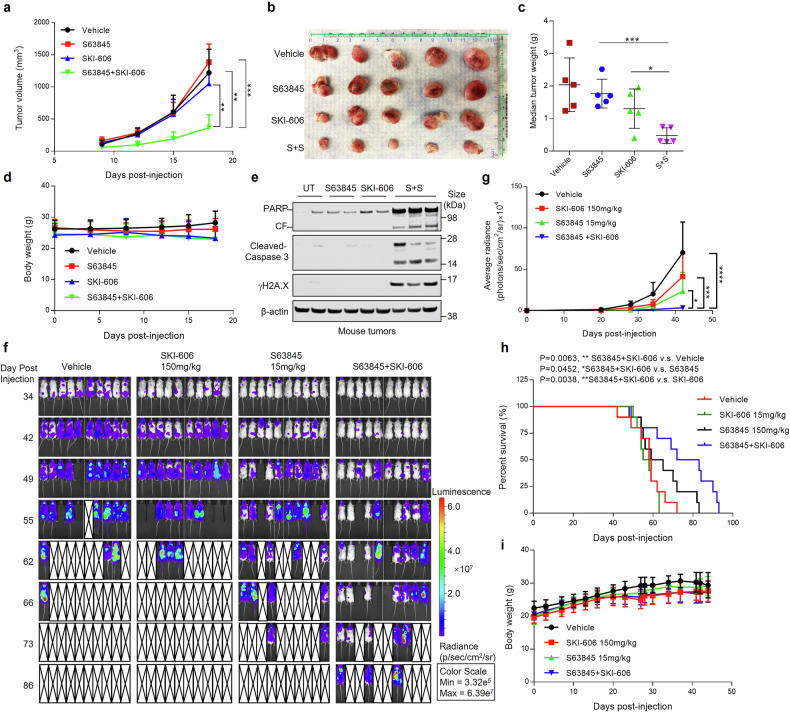
The S63845/SKI-606 regimen exhibits significant in vivo activity. **a**–**e** NOD/SCID-γ NSG mice (5 mice/group) were inoculated in the right flank with 1 × 10^6^ U937 cells. Treatment was initiated after 8 days. S63845 (25 mg/kg, twice a week, I.P.) ± SKI-606 (150 mg/kg, 5 days weekly, p.o.) were administrated weekly for 2 weeks. **a** Tumor size was monitored every other day. Mean tumor volume was calculated using the formula (1/2 × [length × width^2^]). **b**, **c** At day 21, tumors were harvested and weighed. **d** Mouse body weights were monitored every other day throughout the treatment period. *P* > 0.05. **e** Immunoblotting analysis was then performed to monitor expression of PARP, cleaved caspase-3 and γH2A.X. β-actin was assayed to ensure equivalent loading and transfer. **f**–**i** Mice (10 mice/group) were inoculated via tail vein with 5 × 10^6^ MV4-11 cells stably expressing luciferase. After signals were visible (e.g, 13 days after injection of tumor cells), S63845 (15 mg/kg, twice a week for two weeks, then adjusted to one time weekly for three weeks, I.P.) ± SKI-606 (150 mg/kg, 5 days weekly, p.o.) were administrated for 5 weeks. Control animals were administered equal volumes of vehicle. **f** Tumor burden was monitored every week after subcutaneous (sub-Q) injection with 150 mg/kg luciferin using the IVIS 200 imaging system. **g** Quantification of the luminescent signal. Data represents the means ± SD performed on all mice for each group. **h** Kaplan–Meier survival plot (*P* = 0.0452 and 0.0038 for the combination vs S63845 or SKI-606 alone, respectively, by log-rank test). **i** Mouse body weights during treatment (*P* > 0.05). CF, cleavage fragment. *P < 0.05, **P < 0.01, ***P < 0.001, ****P < 0.0001

To assess in vivo anti-leukemic activity of the S63845/SKI-606 regimen, studies were performed in a U937 cell flank tumor model. NSG mice were injected in the right flank with 1 × 10^6^ U937 cells and treated with S63845 25 mg/kg, i.p., two days a week ± SKI-606 150 mg/kg, p.o., five days/week. Tumor volume and body weight were monitored every other day (Fig. 6a–e). Combined treatment markedly reduced tumor volume and weight compared to single agents (Fig. 6a–c) without weight loss (Fig. 6d) or other toxicities. Notably, tumor extracts from mice treated with both agents showed a striking increase in caspase 3/PARP cleavage and γH2A.X generation (Fig. 6e).

Similar results occurred in a luciferase-labeled MV4-11 cell flank model in which mice were treated as outlined in Supplementary Fig. S14a. Combined treatment markedly reduced tumor burden, quantified by photon signals (Supplementary Fig. S14b, c), and dramatically reduced tumor volume (Supplementary Fig. S14d, e). Treatment was unassociated with weight loss (Supplementary Fig. S14f), and tumors extracted from animals receiving both agents displayed marked reductions in MCL-1 expression and increased caspase 3 cleavage (Supplementary Fig. S14g).

Parallel studies were performed in a systemic luciferase-labeled MV4-11 model in which animals were inoculated intravenously (IV) with 5 x 10^6^ cells and treated with S63845 ± SKI-606 as described in the legend (Fig. 6f–i). Tumor burden was monitored weekly with an IVIS imaging system. S63845/SKI-606 co-administration significantly (*P* < 0.05 vs. single agents) reduced leukemia cell burden in vivo whereas single agents exhibited only modest activity (Fig. 6f, g). Survival was significantly prolonged in the combination treatment arm compared with placebo and single-agent arms, and a clear survival increase was apparent after 62 days treatment (Fig. 6f, h). Combined treatment did not induce weight loss or other toxicities (Fig. 6i).

### The MCL-1/SFK inhibitor regimen markedly inhibits leukemic cell expansion in an AML patient-derived xenograft (PDX) model

**Fig. 7:**
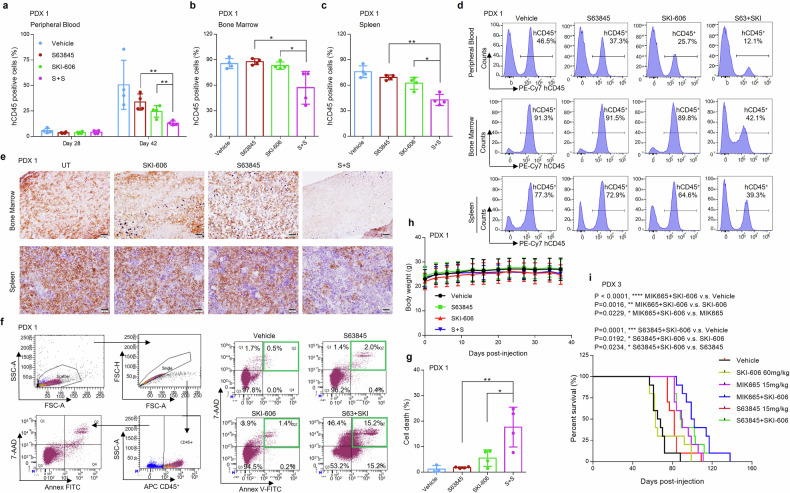
Combined MCL-1 inhibitor/SKI-606 exposure inhibits leukemic cell expansion in an AML Patient-Derived Xenograft (PDX) model. **a**–**h** NOD/SCID-gamma SCF/GM-CSF/IL3 (NSG-SGM3) mice (4 mice/group) were inoculated via tail vein with 0.5 × 10^6^ primary patient-derived AML cells. Treatment was initiated after 6 days. S63845 (20 mg/kg, twice a week, I.P.) ± SKI-606 (150 mg/kg, 5 days weekly, p.o.) were administrated each week for 5 weeks. Control animals were administered equal volumes of vehicle. Peripheral blood human CD45^+^ (hCD45^+^) cells were monitored every two weeks. **a** hCD45^+^ cells in the peripheral blood were quantified by flow cytometry. Data are shown as the mean % ± SD. **b**, **c** After 5-weeks of treatment, mice were sacrificed and hCD45^+^ cells in the bone marrow and spleen were quantified by flow cytometry. **d** The percentage of hCD45^+^ cells in the peripheral blood, bone marrow and spleen in different groups is illustrated in the histogram. **e** IHC of bone marrow (femur) and spleen, stained with monoclonal antibodies for hCD45 in experimental mice. Scale bar = 50 μm. **f** The percentage of cell death in hCD45^+^ cell population in different groups is reflected in the dot plots. **g** Cell death was assessed selectively in the hCD45^+^ cell population by Annexin V/7-AAD staining. **h** Body weights during treatment were monitored twice/week (*P* > 0.05). **i** Kaplan–Me**i**er survival plot. Mice (10 mice/group) were inoculated with 5 × 10^6^ primary blasts #03 via tail vein. *P < 0.05, **P < 0.01

Studies were then extended to PDX models. The clinical features of patient specimens used for PDX models are listed in Supplementary Table S3. Prior to experiments, the assessment of cell engraftment of a patient-derived primary AML specimen (#01) was documented in immunocompromised mice (Supplementary Fig. S15a). Subsequently, the combination treatment strategy was evaluated in a PDX model (Supplementary Fig. S15b). Specifically, NOD/SCID-gamma Il3- GM-SF mice (NSG-SGM3 mice) were inoculated via tail vein with 0.5 x 10^6^ primary blasts, and treatment initiated after 6 days when human CD45^+^ (hCD45^+^) cells were stably detected in the peripheral blood. Animals received S63845 20 mg/kg twice a week i.p. ± SKI-606 150 mg/kg 5 days weekly p.o. for 5 weeks (Supplementary Fig. S15c). The mice were then euthanized, and hCD45^+^ cells monitored in the peripheral blood (PB), bone marrow (BM), and spleen. A significant reduction in hCD45^+^ cells in each of these compartments following combined treatment is shown in Fig. 7a–d. Histopathologic specimens from marrow and spleen showed a dramatic diminution of hCD45^+^ cells in the former, and a marked reduction in the latter with combined drug exposure (Fig. 7e). Compatible results were obtained when cell death of hCD45^+^ marrow cells was determined by flow cytometry, reflected by Annexin V/7-AAD uptake (Fig. 7f, g). Notably, SKI-606 ± S63845 treatment failed to induce weight loss or other toxicities (Fig. 7h). Similar results e.g., diminished hCD45^+^ cells in the PB, BM, and spleen (Supplementary Fig. S16b–e), and the absence of weight loss (Supplementary Fig. S16f) were observed in a second PDX model (Supplementary Fig. S16a) in which mice were treated with SKI-606 + S63845 as above.

Finally, a PDX Kaplan-Meier model was employed to characterize the impact of SKI-606 and MIK665 or S63845 on survival of animals inoculated with a third primary AML (Supplementary Fig. S17a). Kaplan–Meier survival plots revealed very significantly increases in survival for animals receiving SKI-606 with either S63845 or MIK665 compared to single agents or controls (Fig. 7i). Combined regimens resulted in reductions in hCD45^+^ leukemic cells in the peripheral blood (Supplementary Fig. S17b), and were unassociated with weight loss or other toxicities (Supplementary Fig. S17c), arguing that combined Src and MCL-1 disruption exhibits marked anti-leukemic activity in PDX models.

### Omics analysis

The preceding studies established that Src inhibitors blocked MCL-1 antagonist-mediated MCL-1 up-regulation in AML cells through multiple mechanisms, and that these events contributed functionally to synergistic interactions between these agents. However, it remains possible that factors in addition to MCL-1 down-regulation contributed to the pronounced induction of AML cell death by the Src/MCL-1 inhibitor regimen. Previous studies have established the feasibility of employing omics analysis to interrogate interactions between anti-neoplastic agents.^37,38^ To test this possibility, proteomic, phosphorylation array, and metabolomic analysis of cells exposed to these agents alone or together was undertaken, with the goal of identifying perturbations primarily observed in cells exposed to combined therapy.

### Proteomic analysis of MCL-1/Src inhibitor synergism

For these studies, U937 cells were exposed (24 h) to 20 nM S63, 2 µM SKI, or the combination as described above, after which analysis was performed. A volcano plot highlights differentially regulated proteins across each treatment group compared to controls (UT) (Supplementary Fig. S18a). Venn diagrams summarizing these differentially regulated proteins are illustrated in Supplementary Fig. S18b, revealing that combined S63/SKI treatment uniquely induces 45 up-regulated and 23 down-regulated proteins (*p* < 0.05). Reactome pathway enrichment analysis revealed that 45 up-regulated proteins only found in the S63 + SKI treatment group are primarily enriched in the PINK1-PRKN mediated mitophagy (REAC: R-HAS-5205685, adj *p*-value = 5.939 × 10^−3^) and the generic mitophagy pathways (REAC: R-HAS-5205647, adj *p*-value = 1.119 × 10^−2^). Up-regulated proteins involved in these pathways included MFN2, MTERF3, and TOMM20. Conversely, the 23 down-regulated proteins specific to the S63 + SKI treatment group identified 4 proteins (GSK3β, GYG1, PPP2CA, and PPP2R5E) involved in multiple pathways, including glycogen synthesis and degradation, AXIN signaling, APC signaling, AMER1 signaling, and CTNNB1 signaling (Supplementary Fig. S18c). Additionally, STRING analysis indicated that the down-regulated proteins PPP2R5E and PPP2CA have significant connections to BCL2 signaling,^39^ demonstrating high-confidence interactions (score = 0.700) (Supplementary Fig. S18d). Together, these findings raise the possibility that perturbations in these proteins and their associated pathways may contribute to the anti-AML action of combined Src/MCL-1 inhibitor exposure.

### Phosphorylation array analysis of MCL-1/Src inhibitor synergism

A heatmap of the phosphorylation state of 1471 substrates under different treatment conditions is illustrated in Supplementary Fig. S19a. As shown, different substrate phosphorylation levels within the array were detected between groups, and the distribution of differentially (up and down) phosphorylated substrates for S63, SKI, and S63 + SKI groups compared to controls is summarized in the Venn diagrams (Supplementary Fig. S19b). Utilizing pathway databases (KEGG and REACTOME), phosphorylated substrates were clustered into 273 pathways; significantly modified (up and down) pathways are highlighted in Supplementary Fig. S19c. We then analyzed the active and inactive pathways for the combined S63 + SKI group versus those of S63 and SKI alone groups (supplementary Table S4). Notably, activation of the VEGF-related pathway was particularly pronounced in the combination group (Supplementary Fig. S19d). Finally, the activation level of kinases was examined. We estimated the activity scores of 185 kinases for each treatment group and compared results obtained in the combined S63 + SKI group to those in the S63 and SKI groups. We found a significant increase in the activity of kinases LIMK1, MAPK9 and CHEK1, while the activities of the kinases FER and MAPKAPK5 were markedly decreased in the combination group compared to the single agents (Supplementary Fig. S19e). Together, these findings raise the possibility that alterations in the activation status of VEGF-related pathways as well as kinases e.g., MAPK9, FER and MAPKAPK5 may contribute to the anti-AML activity of combined Src/MCL-1 inhibitor exposure.

### Metabolomics profiling reveals the impact of combination treatment on cell metabolism

Links between MCL-1 and hexose kinase 2 (HK2) suggest potential metabolic outcomes for inhibiting MCL-1.^40,41^ On one hand, MCL-1 interacts with HK2 at mitochondria to stimulate glycolysis^41^; on the other hand, this interaction has been proposed as a scaffolding reaction to stabilize MCL-1, independently of HK2 catalytic capability and glucose metabolism per se.^40^ To test whether combined inhibition of MCL-1 and SRC induced specific metabolic changes that may influence outcomes of combinatorial treatment, untargeted metabolomics analysis was performed. Cells were treated with S63, SKI, or the combination (S63 + SKI), and metabolomes were compared to untreated cells. A two-component principle component analysis (PCA) was conducted to assess the differential impact of individual vs. combination treatment. This revealed a pronounced separation between untreated, SKI, S63 and S63 + SKI treated cells (Supplementary Fig. S20a). Furthermore, treatment with both drugs also clearly yielded a pattern distinct from that of either drug alone. Heat mapping further emphasized the relationships between groups, with the combined treatment group exhibiting more intense and on occasion disparate behavior compared to control and single treatment groups (Supplementary Fig. S20b). Synergistic actions of combined treatment was observed by comparing changes in metabolite intensity for statistically significant differences between treatment and control groups. Specifically, S63 and SKI alone induced relatively modest changes in the metabolome compared to the non-additive changes observed in the double treatment group (Supplementary Fig. S20c). The most significantly impacted metabolites included glycolytic intermediates, fructose 1,6-bisphosphate, glyceraldehyde-3 phosphate, and 3-phosphoglyceric acid, all of which increased to a greater extent in the combined treatment groups compared to either group alone (Supplementary Fig. S20d). Consistent with this finding, pathway analysis revealed enrichment of glycolysis/gluconeogenesis in combined-treated vs. either treatment alone, in addition to numerous other changes in metabolic pathways (Supplementary Fig. 20e). Together, these alterations in glucose metabolism and adjacent pathways raise the possibility that metabolic changes induced by combined treatment may contribute to the synergism observed between the two agents.

## Discussion

The anti-apoptotic protein MCL-1 has been implicated in leukemogenesis^42^ and plays a role in the survival of leukemia stem cells,^43^ making it an attractive target in AML. The abundance of MCL-1 is regulated at multiple levels, including transcription, translation, and post-translational events.^44^ These considerations have prompted development of multiple BH3-mimetic MCL-1 antagonists including S63845, AZD5991, MIK665, and AMG 176, among others.^45^ However, such BH3-mimetics, as well as others targeting alternative BCL-2 family proteins e.g., BCL-2 and BCL-xL, elicit a rebound up-regulation of these proteins,^29^ thereby attenuating anti-apoptotic activities. Until recently, the mechanism(s) underlying such events remained uncertain. However, a recent study by Tantawy et al., showed that in CLL cells, MCL-1 antagonists disrupted MCL-1-related ubiquitin ligases, thus disabling MCL-1 degradation and promoting its accumulation.^30^ Whether an analogous mechanism is operative in AML cells remains to be determined. Strategies capable of disrupting this compensatory process and potentiating MCL-1 antagonist-mediated anti-leukemic effects have not yet been explored in any cell type.

The present results argue that several tyrosine kinase inhibitors (TKIs) with known Src inhibitory activities e.g., bosutinib, dasatinib, effectively block MCL-1 antagonist-mediated MCL-1 up-regulation, and in so doing, synergistically potentiate the activity of these agents against AML cells. While such TKIs have primarily been used in the setting of BCR/ABL^+^ leukemias,^46^ Src promotes AML cells survival, and Src inhibitors have demonstrated anti-AML activity in pre-clinical studies.^24^ While the possibility that the TKI-related inhibitory activity of these agents contributed to the prevention of MCL-1 accumulation cannot be ruled out, the finding that Src knock-down recapitulated the effects of pharmacologic agents (e.g., MCL-1 down-regulation and increased apoptosis) argues strongly that Src disruption plays a significant functional role in these events. Analogously, the observation that ectopic MCL-1 expression substantially reduced the lethal effects of MCL-1 antagonist/Src inhibitor regimens further supports the notion that prevention of MCL-1 accumulation plays a key role in drug interactions.

The bulk of evidence suggests that mechanisms by which Src inhibitors block MCL-1 accumulation in cells exposed to MCL-1 antagonists are multi-factorial. In the recent study involving CLL cells,^30^ the ability of MCL-1 antagonists to induce MCL-1 stabilization was linked to multiple factors, including de-ubiquitination, destabilization of the Mule E3-ligase, and dissociation of MCL-1/NOXA interactions.^30^ Here, we found that in AML cells exposed to MCL-1 antagonists/SKI-606 exhibited a marked increase in the association of MCL-1 and K48 ubiquitination, a known degradative process.^47^ The mechanism by which Src interruption promotes degradative ubiquitination of MCL-1 remains to be defined. Notably, combined Src and MCL-1 antagonist exposure dramatically increased the expression of NOXA, which represents an established trigger for MCL-1 degradation.^34^ The ability of NOXA knock-down to attenuate MCL-1 down-regulation as well as Src/MCL-1 inhibitor anti-leukemic activity argues that NOXA up-regulation plays an important functional role in Src/MCL-1 inhibitor interaction. Furthermore, consistent with evidence implicating BAX/BAK interactions in MCL-1 stabilization in CLL cells,^30^ the finding that BAX/BAK knock-out cells were largely protected from the Src/MCL-1 inhibitor regimen strongly implicates these multi-domain proteins in potentiation of MCL-1 antagonist activity in AML cells by Src inhibition.

In addition to these mechanisms, the abundance of MCL-1 is also regulated at the transcriptional level by transcription factors such as STAT3.^44^ MCL-1 is a well-described target of STAT3 action,^48^ as are c-MYC^48,49^ and BCL-xL.^48^ Moreover, STAT3 itself is regulated at the post-translational level by phosphorylation e.g., at Ser727 and Tyr705 residues.^50^ Notably, STAT3 phosphorylation can be induced by Src.^51^ Interestingly, each of these STAT3 target proteins was down-regulated by the SKI-606/MCL-1 inhibitor regimen in association with pronounced STAT3 dephosphorylation at both sites. Moreover, the ability of ectopic expression of BCL-xL and c-MYC to limit regimen activity argues that down-regulation of these proteins contributes functionally to the enhanced induction of cell death. Given these findings, it is tempting to speculate that dephosphorylation/deactivation of STAT3 by Src inhibition contributes to down-regulation of multiple proteins e.g., MCL-1, BCL-xL, and c-MYC implicated in AML cell survival and proliferation.^45,52^

Notably, the MCL-1/Src-inhibitor regimen was toxic to primary, patient-derived AML cells, including populations enriched for more primitive progenitors. Interestingly, several of the mechanisms identified in cell lines such as down-regulation of MCL-1 and dephosphorylation of STAT3 were recapitulated in primary leukemic blasts. However, in contrast to findings in leukemic cells, identical regimens were relatively sparing toward normal hematopoietic progenitor cells (e.g., CD34^+^). One plausible explanation for these disparate responses is that neoplastic cells in general^53^ and human myeloid leukemia cells in particular^54^ are dependent upon STAT3 for survival and proliferation. If this is the case, it would be interesting to determine whether AML cells with high basal levels of STAT3 activation, and presumably addicted to this transcription factor, might be particularly susceptible to the MEK1/2/Src inhibitory strategy. Studies designed to test this concept are currently underway. Finally, it has been reported that some MCL-1 antagonists have been associated with cardiotoxicity as an adverse event,^36^ raising the possibility that this phenomenon might be exacerbated by Src inhibitors. However, combining MCL-1 inhibitors with Src antagonists failed to increase in vitro cell death in a human adult ventricular cardiomyocyte cell line. Whether the lack of toxicity of this regimen could be extrapolated to patients receiving these agents in the clinic remains to be determined.

The Src/MCL-1 inhibitor regimen was active in multiple AML xenograft models, significantly prolonging the survival of animals compared to administration of Src and MCL-1 inhibitors individually. Of note, several of the pharmacodynamic events postulated to contribute to Src/MCL-1 inhibitor synergism e.g., MCL-1 down-regulation, dephosphorylation of STAT3, activation of caspases were recapitulated in leukemic cells extracted from mice treated with both MCL-1 and Src antagonists. Importantly, combined treatment was well tolerated and significantly improved outcomes in three AML PDX model systems. The excellent in vivo tolerability of the regimen is compatible with the notion that this strategy selectively targets neoplastic cells, possibly a consequence of their addiction to MCL-1^12^ and STAT3.^54^

While it is likely that Src and MCL-1 inhibitor interactions stem from MCL-1 down-regulation, it remains possible that perturbations in other proteins contribute to these interactions. For example, proteomic analysis revealed down-regulation of PPP25RE and PP2CA, two proteins known to interact with members of the BCL-2 family,^39^ was particularly marked in cells treated with both agents. Notably, phosphorylation array analysis revealed the pronounced activation of VEGF-related pathways and alterations in the activation status of kinases MAPK9, FER and MAPKAPK5 in the combined treatment group. Similarly, changes in the activity of glycolytic pathways were very pronounced in cells exposed to both agents. It is noteworthy that hexose kinase, a key component of glycolytic pathways, has been shown to serve as a scaffold protein that binds to MCL-1,^40,41^ potentially modifying its disposition or abundance. However, the relative functional contribution of these events compared to the mechanisms described previously (e.g., MCL-1 down-regulation) remains to be determined. Studies addressing these issues are currently in progress.

**Fig. 8:**
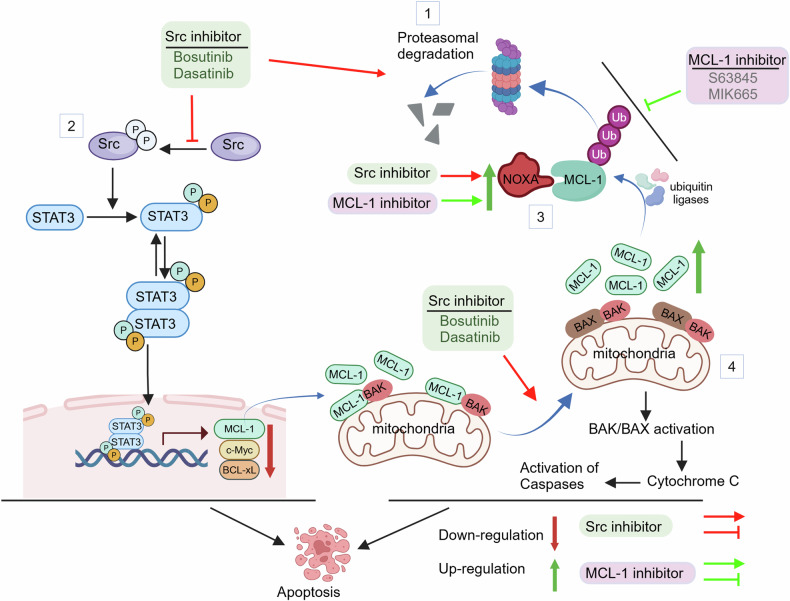
Schematic model of MCL-1/SRC inhibitor interactions in AML cells. MCL-1 antagonists, through a yet-to-be-determined mechanism, disrupt degradative MCL-1 ubiquitination, leading to MCL-1 stabilization, accumulation, and prevention of apoptosis. This phenomenon is antagonized by Src inhibitors, resulting in down-regulation of this anti-apoptotic protein (1). In addition, Src inhibitors counteract the activating phosphorylation of the STAT3 trasnscription factor, thereby inhibiting the transcription of multiple genes implicated in AML cell survival and proliferation e.g., BCL-xL, c-MYC, and MCL-1 (2). Moreover, combining Src and MCL-1 inhibitors leads to up-regulation of NOXA, a well-established promoter of MCL-1 degradation (3). Collectively, these events lead to dissociation of MCL-1 from BAX and BAK, thereby activating these proteins and triggering mitochondrial injury and apoptosis (4)

In summary, the results of the present study indicate that in AML cells, MCL-1 antagonists trigger a rebound stabilization and accumulation of this protein by disrupting its degradative ubiquitination, leading to attenuation of cell death.^30^ Importantly, Src inhibitors oppose this process, resulting in MCL-1 down-regulation and highly synergistic anti-leukemic effects. A summary of the multiple mechanisms contributing to this interaction is shown in Fig. 8. According to this model, Src inhibitors, through a yet to be determined mechanism, promote degradative ubiquitination of MCL-1, preventing the stabilization and accumulation of this anti-apoptotic protein. Additionally, Src inhibitors oppose activating phosphorylation of the STAT3 transcription factor, which inhibits the transcription of multiple genes that encode protein implicated in AML survival and proliferation e.g., MCL-1,^18^ BCL-xL,^52^ and c-MYC.^55^ Furthermore, combining MCL-1 with Src inhibitors leads to pronounced up-regulation of NOXA, a well-established promoter of MCL-1 degradation.^34^ Collectively, these pharmacodynamic events trigger a marked potentiation of BAX/BAK-mediated cell death and synergistic anti-AML activity. Finally, it is possible that perturbations in proteomic and glycolytic pathways contribute to the marked increase in cell death. Importantly, these interactions occur in primary AML cells but not in their normal counterparts, and the Src/MCL-1 antagonist strategy is well tolerated and effective in multiple xenograft and PDX AML models. Given the approval of TKI/Src inhibitors in the clinic,^56^ as well as the ongoing evaluation of MCL-1 antagonists in leukemia and multiple other malignancies,^18^ the Src/MCL-1 strategy may warrant attention in AML and potentially other neoplastic disorders.

## Materials and methods

### Cell lines and reagents

Human leukemia U937 and MV4-11 cells were purchased from the ATCC (Manassas, Virginia, USA), MOLM-13 and OCI-AML3 cells were purchased from DSMZ (Brunswick, Lower Saxony, Germany). All cell lines were cultured as described earlier.^57^ Human cardiomyocyte AC16 cells were purchased from the ATCC, and maintained in DMEM/F12 containing 2mM L-Glutamine supplemented with 12.5% FBS and penicillin-streptomycin. MycoAlert (Lonza, Allendale, NJ) Mycoplasma assays were performed multiple times during this study. All experiments utilized logarithmically growing cells ((3–4) × 10^5^ cells/ml). Various stable or knock down cell lines used in this study are described in Supplementary Methods.

For information regarding S63845 (S63), MIK665 (MIK), Bosutinib/SKI-606 (SKI), Dasatinib (Das), and other reagents, Kits and plasmids, see Supplementary Table S1. All drugs were dissolved in DMSO, aliquoted, and stored at −80 °C. In all experiments, final DMSO concentrations did not exceed 0.1%. Drug concentrations used in cytotoxicity studies were selected based on relatively modest (e.g., generally <20%) single-agent induction of cell death.

### Isolation of patient-derived leukemic blasts and normal CD34^+^ cells

Bone marrow or peripheral blood from patients with AML were obtained with written informed consent from all patients. These studies were conducted in accordance with recognized ethical guidelines (e.g., the Declaration of Helsinki). Mononuclear cells were isolated using the Ficoll Histopaque method (#10771, Sigma-Aldrich, USA). Normal hematopoietic CD34^+^ cells were isolated from human umbilical cord blood obtained from patients undergoing normal deliveries. Isolated cells were cultured in RPMI1640 medium containing 10% FBS at a concentration of 1 × 10^6^ cells/ml, and exposed to inhibitors as described in the Results section. All studies were sanctioned by the Investigational Review Board of Virginia Commonwealth University (#MCC-8712-3A; MCC-02447; MCC-03340).

### Patient-derived xenograft (PDX) models of AML

All animal studies were performed under protocol AM10204 with approval from the Institutional Animal Care and Use Committee, and in strict accordance with the guidelines of AAALAC, USDA, and PHS. S63845 was prepared by dissolving the drug in 2% kolliphore and 98% sterile PBS; MIK665 was provided by Novartis and prepared strictly according to the instructions. SKI-606 was dissolved in 0.5% methylcellulose and 0.4% polysorbate 80 (Tween 80). NOD/SCID-gamma Il3- GM-SF mice (NSG-SGM3; Jackson Laboratories, Bar Harbor, ME, USA) were used for the following models: (1) To establish mouse models of primary patient AML, 0.5 × 10^6^ leukemic blasts #01 or 5 × 10^6^ leukemic blasts #02 were injected into 6-week old mixed sex NSG-SGM3 mice via tail-vein injection. Animals were monitored for leukemia progression using flow cytometric analysis of peripheral blood for hCD45^+^ cells. After 6 days (Pt #01) or 12 days (Pt #02), the mice were divided into treatment groups (4 mice/group) and received either S63845 (20 mg/kg, 2 days per week, intraperitoneal), SKI-606 (150 mg/kg, 5 days per week, oral), or a combination of both treatments for 5 weeks (#01) or 9 weeks (#02). Control mice were administered equal volume of vehicle. Subsequently, the mice were euthanized, and the percentage of hCD45^+^ cells in the bone marrow and spleen was assessed by flow cytometric analysis and IHC staining. (2) For survival studies, mice (9 mice/group) were inoculated with 5 × 10^6^ primary AML blasts #03 via the tail vein. After one week, the mice were assigned to treatment groups and received S63845 (15 mg/kg, 2 days per week, intraperitoneal) ± SKI-606 (60 mg/kg, 5 days per week, oral) or MIK665 (15 mg/kg, 2 days per week, intraperitoneal) ± SKI-606 (60 mg/kg, 5 days per week, oral) for 8 weeks. Control mice received an equivalent volume of vehicle. AML growth was monitored every 2 weeks by assessing the percentage of hCD45^+^ cells in peripheral blood through flow cytometry, and body weight were monitored every other day throughout the study to monitor toxicity. The duration of treatment was guided by drug tolerability and effects on tumors.

### Statistical analysis

Values represent the means ± standard deviation (SD) for at least three independent experiments performed in triplicate. Statistical significance for in vitro studies was determined by using the Student *t* test or one-way analysis of variance with the Tukey-Kramer multiple comparisons test. Drug synergism was determined by median dose-effect analysis using Calcusyn software.^58^ Survival rates were determined by the Kaplan–Meyer analysis, and comparisons of survival curves and median survival were analyzed by the log-rank test. The significance of *P* values is indicated as follows: *P < 0.05; **P < 0.01; ***P < 0.001; ****P < 0.0001; ^#^*P* < 0.05; ^##^*P* < 0.01; ^###^*P* < 0.001; ^####^*P* < 0.0001.

For additional information, see Supplementary Materials and Supplementary Table S1.

## Supplementary information

Supplemental Material

Supplementary Table S1

Supplementary Table S2

Supplementary Table S3

Supplementary Table S4

Supplemental Material Original blot

## Data Availability

All data needed to evaluate the conclusions in the article are present in the article and/or the Supplementary Materials. The data and materials used in the current study are available from the corresponding authors upon reasonable request. All original source data (chiefly western blot data) linked to the figures in the manuscript can be downloaded at the website OSFHOME: https://osf.io/h6n5m/?view_only=7f6a6c337834455e96c47c5ee0853d22.

## Materials availability

Materials used in the current study are available from the corresponding authors upon reasonable request.
