# Supplementary material for: Src inhibition potentiates MCL-1 antagonist activity in acute myeloid leukemia
Source: Signal Transduct Target Ther. 2025 Feb 10;10:50. doi: 10.1038/s41392-025-02125-x (PMC11808118; doi:10.1038/s41392-025-02125-x)
Supplement: Supplementary file 1 — Supplemental Material [file 41392_2025_2125_MOESM1_ESM.docx]

Supplementary Materials for

**Src inhibition potentiates MCL-1 antagonist activity in AML**

Xiaoyan Hu^1^, Lin Li^1^, Jewel Nkwocha^1^, Maciej Kmieciak^2^, Shengzhe Shang^2^, L. Ashley Cowart ^2, 3^, Yang Yue^4^, Katsuhisa Horimoto^5^, Adam Hawkridge^6^, Arjun Rijal^2^, Adolfo G. Mauro^7^, Fadi N. Salloum^7^, Lori Hazlehurst^8^, Konstantinos Sdrimas^9^, Zackary Moore^2^, Liang Zhou^1, 10^, Gordon D. Ginder^1, 2^, Steven Grant^1, 2^†.

Correspondence to: [steven.grant@vcuhealth.org](mailto:steven.grant@vcuhealth.org)

**This PDF file includes:**

Materials and Methods

Supplementary Figures. S1 to S20

**Other Supplementary Materials for this manuscript include the following:**

Supplementary Tables. S1 to S4

Supplementary Table S1. Information for kits, reagents, plasmids, shRNA and antibodies used in this study.

Supplementary Table S2. Clinical, molecular, and cytogenetic characteristics of patient samples exposed to MCL-1 inhibitors ± SKI-606.

Supplementary Table S3. Clinical, molecular, and cytogenetic characteristics of patient samples used for PDX models.

Supplementary Table S4. List of active and inactive pathways of (S63+SKI) group compared with those of S63 and SKI groups.

***Materials and Methods***

**Cell lines and reagents**

Knockdown of Src, MCL-1 or NOXA proteins were accomplished by infecting U937and MV4-11 cells with lentiviruses carrying specific pLKO.1 shRNA construct (Open Biosystems, Huntsville, AL) as previously described^1^. U937 and MV4-11 cells lacking expression of BAX, BAK or BAX/BAK were generated using a CRISPR-Cas9 system as previously described^2^. U937 and MV4-11 cells ectopically expressing MCL-1 and BCL-xL were generated as previously described^3^. STAT3-CA cells was selected in U937 and MV4-11 cells infected with a lentivirus harboring constitutively-active STAT3 (Addgene, #24983) as before^4^. pMYC-GFP (Addgene, # 42142) plasmids were transfected with a Nucleofector Device into U937 cells following the protocol of Amaxa Cell Line Nucleofector^TM^ Kit C (Lonza, VCA-1004). For information regarding plasmids, see Supplementary Table S1.

**Cell viability assay**

Cell proliferation was determined by CellTiter‐Glo® luminescence cell viability assay (Cat# G7570, Promega, Madison, WI, US) in accordance to the manufacturer’s instructions.

**Analysis of cell death**

The extent of cell death was routinely assessed by a 7-AAD staining assay as previously described^5^.

For AML primary cell analysis, mononuclear cells isolated from AML patient bone marrows and peripheral blood were blocked with TruStain FcX (BioLegend, Cat# 422302) on ice for 5min, stained with CD45-APC-FireTM 750, CD34-PE, CD38-PE/Cy7 and CD123-APC (Biolegend, San Diego, CA) on ice for 20~30min followed by staining with Annexin V- FITC and 7-AAD at room temperature in the dark for 15min. The percentage of apoptotic (Annexin V+) cells in the CD45dim SSlow/CD34+ and CD45dim SSlow/CD34+/CD38−/CD123+ population was determined using a FACS Canto flow cytometer (BD Biosciences).

**Subcellular fractionation**

Cytoplasmic and nuclear proteins were extracted using Active Motif Nuclear Extract Kit (Cat# 40010, Carlsbad, CA) protocol and reagents (PBS, Phosphatase inhibitors, Hypotonic buffer, DTT, Lysis Buffer AM1, and Protease inhibitor cocktail).

**BAX and BAK conformational change**

BAX and BAK conformational change were assessed as previously described^1^. The antibody BAK AB-1 (Cat #AM03100UG) was purchased from Thermo Fisher Scientific. BAX 6A7 antibodies (Cat #sc-23959) were purchased from Santa Cruz Biotechnology.

**Quantitative Real-time PCR**

Total RNA was extracted with the RNeasy Plus Mini Kit (Qiagen #74134) from AML cell lines. 2µg RNA was reverse-transcribed by RevertAid First Strand cDNA Synthesis Kit (Thermo Fisher #K1622) according to the manufacturer’s instructions. Using Taqman Gene Expression Assay probe/primer [Hs03043899_m1 for MCL-1] and *Taq* DNA polymerase reaction buffer (Thermo Fisher #18067-017), cDNAs were amplified in a fluorescence thermocycler (ABI StepOnePlus Real-time PCR System, Applied Biosystems, CA, USA) and were analyzed based on the expression level of GADPH with SDS2.2 software (Applied Biosystems).

**Cytosolic fractionation**

Cytosolic fractions were separated following a previously published method^6^. In brief, cells were lysed using digitonin buffer (150 mM NaCl, 50 mM HEPES pH 7.4, 25ug/ml digitonin, proteinase cocktail) on ice for 10 min, after which cytosolic fractions were separated by centrifugation at 2,000 RCF for 2 min. Proteins were analyzed by western blot to evaluate cytochrome c, BAK, BAX release into the cytosol.

**Immunoprecipitation and Immunoblotting**

For immunoprecipitation, cells were lysed in buffer containing 25 mM Tris•HCl pH 7.4, 150 mM NaCl, 1% NP-40, 1 mM EDTA, 5% glycerol and protease inhibitor Cocktail (Thermo scientific #78440) after which 500 μg of protein lysate was subjected to immunoprecipitation using the designated antibodies: BAK (#517390, Santa Cruz) and MCL-1 (#559027, BD Pharmingen). Immunoblotting was performed using the immunoprecipitates or whole cells lysates as previously described in detail^7^. The primary antibodies used in this study were as follows: rabbit anti- cleaved PARP (Cell Signaling Technology Cat# 9541), rabbit anti-PARP (Cell Signaling Technology Cat# 9532), rabbit anti-γH2A.X (Cell Signaling Technology Cat# 2577), rabbit anti-cleaved Caspase-3 (Cell Signaling Technology Cat# 9661), rabbit anti-STAT3 (Cell Signaling Technology Cat# 12640), rabbit anti-phospho-STAT3 (Tyr705, Cell Signaling Technology Cat# 9145), rabbit anti-phospho-STAT3 (Ser727, Cell Signaling Technology Cat# 9134), rabbit anti-BCL-xL (Cell Signaling Technology Cat# 2764), rabbit anti-c-Myc (Cell Signaling Technology Cat# 5605), rabbit anti-MCL-1 (Cell Signaling Technology Cat# 94296), rabbit anti-BAK (Cell Signaling Technology Cat# 12105), rabbit anti-BAX (Cell Signaling Technology Cat# 5023), goat anti-NOXA (Santa Cruz Biotechnology, Cat# sc26917), rabbit anti- K48 linkage Specific Polyubiquitin (Cell Signaling Technology Cat# 8081), mouse anti-P84 (Abcam, Cat# ab487), mouse anti-FLAG (Sigma-Aldrich Cat# F1804), rabbit anti-β-actin (Sigma-Aldrich Cat# A2066), mouse anti-GAPDH (Cell Signaling Technology Cat# 97166) and mouse anti-α-tubulin (SA, CP06). The secondary antibodies used were mouse anti-goat IgG-HRP (Santa Cruz Biotechnology, Cat# sc2354), goat anti-mouse (Sera Care KPL Cat# 5450-0011) and goat anti-rabbit (Sera Care Cat# 5220-0458) IgG-peroxidase labeled. Primary antibodies were used at 1:1000 dilutions (Cell Signaling Technology, Abcam), 1:1,000 dilutions (Sigma-Aldri), and 1:200 dilutions (Santa Cruz). Secondary antibodies were used at 1:5000 dilutions (sera care). Images were captured with an Odyssey® Fc Imaging Syetem (LI-COR). Images were quantified and analyzed using ImageJ software.

**Immunofluorescence and flow cytometry using ImageStream**

Cells were fixed in 4% paraformaldehyde for 15 minutes at room temperature, and permeabilized by incubation in 90% methanol on ice for 30 minutes. Cells were incubated with primary antibodies for 1 hour at room temperature: 1:50 anti-phospho-STAT3(Tyr705; Cell Signaling Technology, # 9145), 1:500 anti-phospho-STAT3 (Ser727; Novus, # IC4934U); and secondary antibodies at room temperature, in the dark for 30 minutes: 1:1,000 Alexa Fluor 488 (Life Technologies, # A11070) and Alexa Fluor 647 (Life Technologies, # A21235). Cells were also stained with DAPI 1:100 for 5 minutes before imaging. The cells were washed in 1,000 μL of PBS-2%FBS and recovered by centrifugation at 300 g for 5 minutes; and the incubation buffer for the antibodies was made from 5% BSA and 0.3% TritonX dissolved in PBS. Cells were resuspended in 60 μL of PBS-2%FBS and analyzed with an ImageStream (Amnis) image flow cytometer.

**STAT3 DNA-binding activity**

The DNA binding capacity of STAT3 was determined in cell nuclear extracts using the TransAM® STAT3 activation assay kit (Active Motif #45196 and #40010) according to the manufacturer’s instructions. Resultant absorbance at 450 nm that correlates with STAT binding to a consensus DNA sequence was read using the Promega Glomax Multi Detection Plate Reader (Promega).

**Immunohistochemistry (IHC) staining**

IHC staining was performed with Monoclonal Mouse Anti-Human CD45 (#M0701, dilution 1:200, Dako, Santa Clara, CA) using the Mouse on Mouse (M.O.M.™) solution and ImmPRESS™ HRP Anti-mouse IgG (Peroxidase) Polymer Detection Kits (Vector, Burlingame, CA) as previously described^8^.

**Animal studies**

All animal studies were Institutional Animal Care and Use Committee approved and performed under protocol AM10204 in accordance with AAALAC, USDA, and PHS guidelines. NOD-SCID IL2Rgamma^null^ mice (Jackson Laboratories Bar Harbor, ME, RRID: IMSR_JAX:005557) were used for the flank and systemic models:

1) Flank Model:

Mice were subcutaneously injected with either 1×10^6^ U937 cells or 5×10^6^ luciferase-expressing MV4-11 cells in the flank. When tumors grew to 5 mm (length), mice were subjected to treatment with either S63845 (25mg/kg, 2 days per week, intraperitoneal), SKI-606 (150mg/kg, 5 days per week, oral), or a combination of both treatments for 2 weeks (U937) or 3 weeks (MV4-11). Control animals received an equal volume of vehicle. Tumor growth and body weight were monitored every other day; when tumor size reached 17mm, mice were euthanized. Tumor volumes were calculated using the formula (length x width^2)/2.

2) Systemic Xenograft Model:

Mice were intravenously injected via the tail vein with 5x10^6^ luciferase-expressing MV4-11 cells. One week after cell injection (when luciferase activity was detected), mice were treated with S63845 (15mg/kg, 2 days per week for 2 weeks, followed by adjustment to 1 day per week for 3 weeks, intraperitoneal) ± SKI-606 (150mg/kg, 5 days per week, oral), or vehicle alone. AML tumor growth was monitored using the IVIS 200 imaging system (Xenogen Corporation, Alameda, CA, USA) as previously described^8^ , and body weights were determined every other day throughout the study to monitor toxicity. The duration of treatment was guided by drug tolerability and effects on tumors.

**Proteomics analysis**

U937 cells (3×10^5 cells/ml, 20 ml) were treated in triplicate with 20 nM S63 ± 2 µM SKI for 24 hours, after which cells were harvested and lysed in buffer (50 mM HEPES, pH 8; 150 mM NaCl; 1X protease/phosphatase inhibitors; 0.8% SDS) using a probe sonicator (6 cycles, sec ON/OFF at 40% amplitude). Total protein was determined using the BCA protein assay kit (Thermo, #23225) and a Synergy H1 Reader (Bio-Tek) at 562 nm following a 30 min incubation with shaking at 37°C, with standard curves from 0-2000 µg/ml using lysis buffer as a diluent.

The samples were processed for proteomics analysis using the PreOmics iST sample preparation kit(#P.O.00027) following the manufacturer’s protocol. 80µl of the PreOmics iST LYSE buffer was added to 200µg of protein, incubated at 95°C for 10 minutes with gentle mixing at 1000rpm. Next, 80 µl of the PreOmics iST DIGEST solution was added, followed by a 2-hour incubation at 37°C, 500 rpm. After incubation, 100µl of the PreOmics iST STOP solution was added, and the mixture was centrifuged at 3800rcf for 3min to ensure complete flow through and washed successively with 200µl of WASH 1 and 200µl of WASH 2 solution with centrifugation after each wash. The cartridge was then transferred to a fresh collection tube, and two cycles of 100µl of ELUTE solution was added, centrifuging after each cycle. The total 200µl ELUTE solution was dried using a vacuum evaporator at 45°C and then resuspended in 100 µl of 0.1% formic acid. Peptide concentration was measured using the Fluorescent Peptide Assay kit (Thermo, #23290) and a Synergy H1 Reader (Bio-Tek) at 390/475nm, with standard curves from 0-750µg/ml using 0.1% formic acid as a diluent.

LC-MS/MS analysis were performed using a 480 Exploris tandem mass spectrometer (Thermo) coupled to a Neo Vanquish nanoflow UPLC system (Thermo). Peptides (500 ng) were injected onto the column assembly and eluted with an acetonitrile/0.1% formic acid gradient at a flow rate of 350 nL/min over 2 hours. The nano-spray ion source was operated at 1.9 kV. Data-dependent acquisition (DDA) collected full scan mass spectra (375-1500 *m/z*) at an AGC = 300% and an ITmax = Auto followed by 10 HCD tandem mass spectra per second from 78-6000 *m/z* at an NCE = 30%, AGC = Standard, and ITmax = Auto.

Data Analysis. The data were analyzed in Proteome Discoverer (ver 3.0) using the Sequest HT search algorithm and the Uniprot Human database (download date 01/03/2024). Proteins were identified at an FDR <0.01 and quantification used the peptide intensities. Raw protein abundances were normalized in Proteome Discoverer using the “Total Peptide Abundance” method and ratios were determined using the pair-wise peptide ratio-based method. Statistical significance of differentially expressed proteins is reported as an adjusted *p*-value (Benjamini-Hochberg).

**Phosphorylation array analysis**

Cells were treated with 20 nM S63 ± 2 µM SKI for 24 hours. After treatment, the cells were collected, lysed, and the lysate was applied to a protein array for phosphorylation array analysis. A phosphorylation reaction solution (2.5 mL per array, containing more than 100 µg of proteins) was added directly to the array and incubated for 2 hours at 30°C. After incubation, the solution was replaced with a Termination Buffer (3.0 mL per array) and shaken for 5 minutes. The array was then washed three times with TBS-T (3.0 mL per array) for 10 minutes each, with shaking. Next, the array was soaked in Blocking Buffer (4.0 mL per array) for 30 minutes, followed by another three washes with TBS-T. Subsequently, the array was incubated in Phos-tag solution (4.0 mL per array) for 16 hours at 4°C with shaking in the dark. After additional washing with TBS-T, the array was rinsed twice with purified water and allowed to air-dry. Fluorescence scanning of the proteins on the array was performed using the InnoScan 710 InfraRed AL, and fluorescence intensity was quantified with Mapix image analysis software. This method enabled the simultaneous measurement of the phosphorylation levels of 1,471 substrates across 273 signal transduction pathways.

From the phosphorylation array analysis data, we extracted four types of information using a mathematical platform: differentially phosphorylated substrates (signatures), related pathways, pathway activity, and kinase activity^9^. First, substrate signatures were estimated using three methods, with an integration probability *q* calculated through meta-analysis, establishing a threshold at *q<0.05*. Second, we assessed the proportion of differentially phosphorylated substrates (both up- and down-regulated) within the constituent molecule groups of a proprietary dataset containing 273 pathway groups, independently classified according to KEGG and REACTOME databases. Pathway probabilities were similarly calculated with a threshold of *q<0.1*. Third, pathway activity was evaluated by calculating the consistency of the phosphorylation data with substrate interaction graphs, using a threshold of *p<0.2* to identify active pathways. Finally, kinase activation levels were estimated as activity scores based on a correspondence table linking kinases to their respective substrate group.

**Untargeted metabolomic profile analysis with high resolution LC-MS^2^**

Treated cell samples were collected into cold 80% ethanol, sonicated, and centrifuged 4 °C at 10000 rpm for 15 min to remove proteins. Supernatants were collected and dried down and stored at -80°C until analysis and then reconstituted in 200 µl Optima grade H_2_O for analysis. A pooled quality control (QC) sample was generated with an equal volume of each sample and used to condition the column and to monitor the intensity variation of internal standards. 10µl reconstituted sample was injected each for positive and negative ionization modes. Sample analysis was conducted with Vanquish ultra-high performance liquid chromatography (UHPLC) system coupled to a Q-Exactive HF mass spectrometer (Thermo Fisher Scientific, Waltham, MA, USA) with electrospray ionization source. Acquity high strength silica (HSS) pentafluorophenyl (PFP) columns (150 mm × 2.1 mm × 1.8 µm; Waters) was used to separate the metabolites at 30 °C at a flow rate of 500 µL/min using a mobile phase of 0.1% formic acid in water, v/v (A) and 0.1% formic acid in acetonitrile v/v (B) over a 15-minute (0 min 98% (A), 0-3.5 min 98% (A); 3.5-11.5 min, 75% (A); 11.5-12.5 min, 5% (A); 12.5-15.0 min, 5% (A)). Polar metabolites from each MS^1^ data was collected with full scan with m/z from 60 to 800. Data-dependent fragmentation (ddMS^2^) of the top 5 most abundant peaks were acquired with the pooled QC samples.

Data were pre-processed using peak detection, mass spectral deconvolution, retention time alignment and feature grouping in XCMS^10^ under R (version 4.2). The variation of mass tolerance was set as 5 ppm and retention time tolerance of 0.2 min. The annotation of metabolites was conducted through Compound Discoverer 3.3 (Thermo Fisher Scientific) with ddMS^2^ data. The intensities of the retained features were normalized with MSTUS approaches prior to statistical analysis^11^.

Statistical analysis. Principle component analysis (PCA) of the HR LC-MS data was performed on Metaboloanalyst 6.0^12^. A post-hoc Tukey test (with false discovery rate correction) was used to compare the normalized annotated metabolites in every two groups and the adjusted *p* value of 0.05 was used to identify features with significant differences between groups. Features from positive and negative mode were combined into a single dataset, and duplicates were removed, resulting in a total of 2916 distinct features across all samples.

***References:***

1 Rahmani, M. *et al.* Inhibition of Bcl-2 antiapoptotic members by obatoclax potently enhances sorafenib-induced apoptosis in human myeloid leukemia cells through a Bim-dependent process. *Blood* **119**, 6089-6098(2012).

2 Rahmani, M. *et al.* Cotargeting BCL-2 and PI3K Induces BAX-Dependent Mitochondrial Apoptosis in AML Cells. *Cancer Res* **78**, 3075-3086(2018).

3 Rahmani, M. *et al.* Dual inhibition of Bcl-2 and Bcl-xL strikingly enhances PI3K inhibition-induced apoptosis in human myeloid leukemia cells through a GSK3- and Bim-dependent mechanism. *Cancer Res* **73**, 1340-1351(2013).

4 Zhou, L. *et al.* Chk1 Inhibition Potently Blocks STAT3 Tyrosine705 Phosphorylation, DNA-Binding Activity, and Activation of Downstream Targets in Human Multiple Myeloma Cells. *Mol Cancer Res* **20**, 456-467(2022).

5 Chen, S., Dai, Y., Harada, H., Dent, P. & Grant, S. Mcl-1 down-regulation potentiates ABT-737 lethality by cooperatively inducing Bak activation and Bax translocation. *Cancer Res* **67**, 782-791(2007).

6 Holden, P. & Horton, W. A. Crude subcellular fractionation of cultured mammalian cell lines. *BMC Res Notes* **2**, 243 (2009).

7 Li, L. *et al.* Non-canonical role for the ataxia-telangiectasia-Rad3 pathway in STAT3 activation in human multiple myeloma cells. *Cell Oncol (Dordr)* **46**, 1369-1380 (2023).

8 Satta, T. *et al.* Dual mTORC1/2 Inhibition Synergistically Enhances AML Cell Death in Combination with the BCL2 Antagonist Venetoclax. *Clin Cancer Res* **29**, 1332-1343 (2023).

9 Horimoto, K. *et al.* Phosphorylated protein chip combined with artificial intelligence tools for precise drug screening. *J Biomed Res* **38**, 195-205(2024).

10 Yu, M., Dolios, G. & Petrick, L. Reproducible untargeted metabolomics workflow for exhaustive MS2 data acquisition of MS1 features. *J Cheminform* **14**, 6 (2022).

11 Fu, J. *et al.* Optimization of metabolomic data processing using NOREVA. *Nat Protoc* **17**, 129-151 (2022).

12 Pang, Z. *et al.* MetaboAnalyst 6.0: towards a unified platform for metabolomics data processing, analysis and interpretation. *Nucleic Acids Res* **52**, W398-W406 (2024).

***Supplementary Figures S1-S20***

**Figure S1**


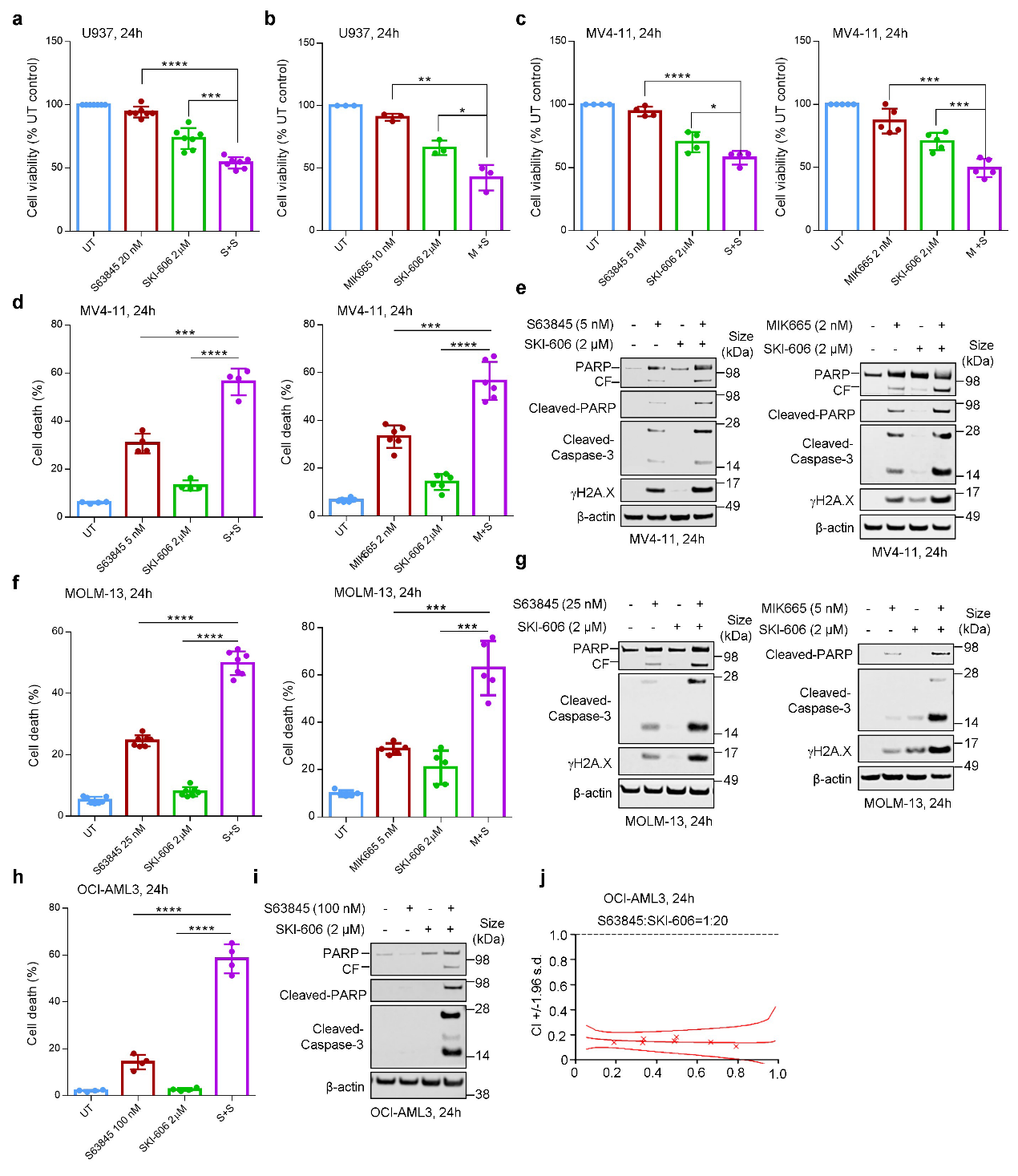


**Supplementary Figure S1**

**MCL-1 inhibitors interact synergistically with SKI-606 to induce apoptosis in multiple AML cells.**

**a-c.** U937 and MV4-11 cells were exposed (24 hours) to indicated concentrations of S63845 or MIK665 ± SKI-606, followed by a CellTiter-Glo® Luminescent assay to monitor cell viability.

**d, f and h.** MV4-11, MOLM-13 and OCI-AML-3 cells were exposed to the indicated concentrations of S63845 or MIK665 ± SKI-606 for 24 hours, followed by flow cytometric analysis of cell death after staining with 7-AAD.

**e, g and i.** Cells were incubated with S63845/MIK665 ± SKI-606 for 24 hours, after which PARP, cleaved-PARP, cleaved-Caspase-3, and γH2A.X were monitored by immunoblotting analysis. β-actin was assayed to ensure equivalent loading and transfer.

**j,** OCI-AML-3 cells were exposed (24 hours) to varying concentrations of S63845 ± SKI-606 at a fixed ratio (S63: SKI=1:20), after which the percentage of 7-AAD^+^ cells was determined. Median dose-effect analysis was then employed to characterize the nature of the interaction between these agents. Combination index values <1.0 denote a synergistic interaction.

All experiments were repeated at least three times. Values shown were mean and SD. CF, cleavage fragment. ***P* < 0.01, ****P* < 0.001, *****P* < 0.0001.

**Figure S2**

**
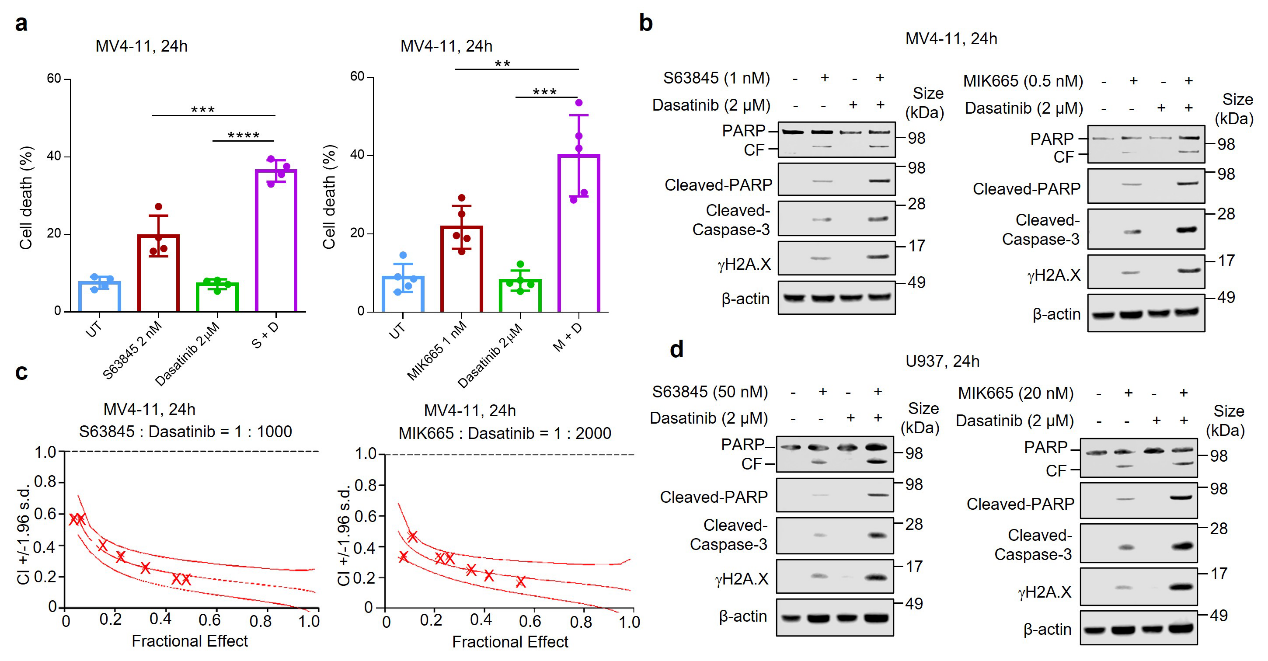
**

**Supplementary Figure S2**

**MCL-1 inhibitors interact synergistically with dasatinib to induce apoptosis in AML cells.**

**a,** MV4-11 cells were exposed to the indicated concentrations of S63845/MIK665 ± dasatinib for 24 hours, followed by flow cytometric analysis of cell death after staining with 7-AAD. Experiments were repeated at least three times. Values shown were mean and SD. ***P* < 0.01; ****P* < 0.001; *****P* < 0.0001.

**b,** Cells were incubated with S63845/MIK665 ± Dasatinib for 24 hours, after which PARP, cleaved-PARP, cleaved-Caspase-3, as well as γH2A.X were monitored by immunoblotting analysis. β-actin was assayed to ensure equivalent loading and transfer.

**c,** Cells were exposed (24 h) to varying concentrations of S63845/MIK665 ± dasatinib at a fixed ratio (S63: das=1:1000 and MIK: das=1:2000), after which the percentage of 7-AAD^+^ cells was determined. Median dose-effect analysis was then employed to characterize the nature of the interaction between these agents. Combination index values <1.0 denote a synergistic interaction.

**d,** U937 cells were incubated with S63845/MIK665 ± Dasatinib for 24 hours, after which western blot assays were performed as in **b**.

**Figure S3.**


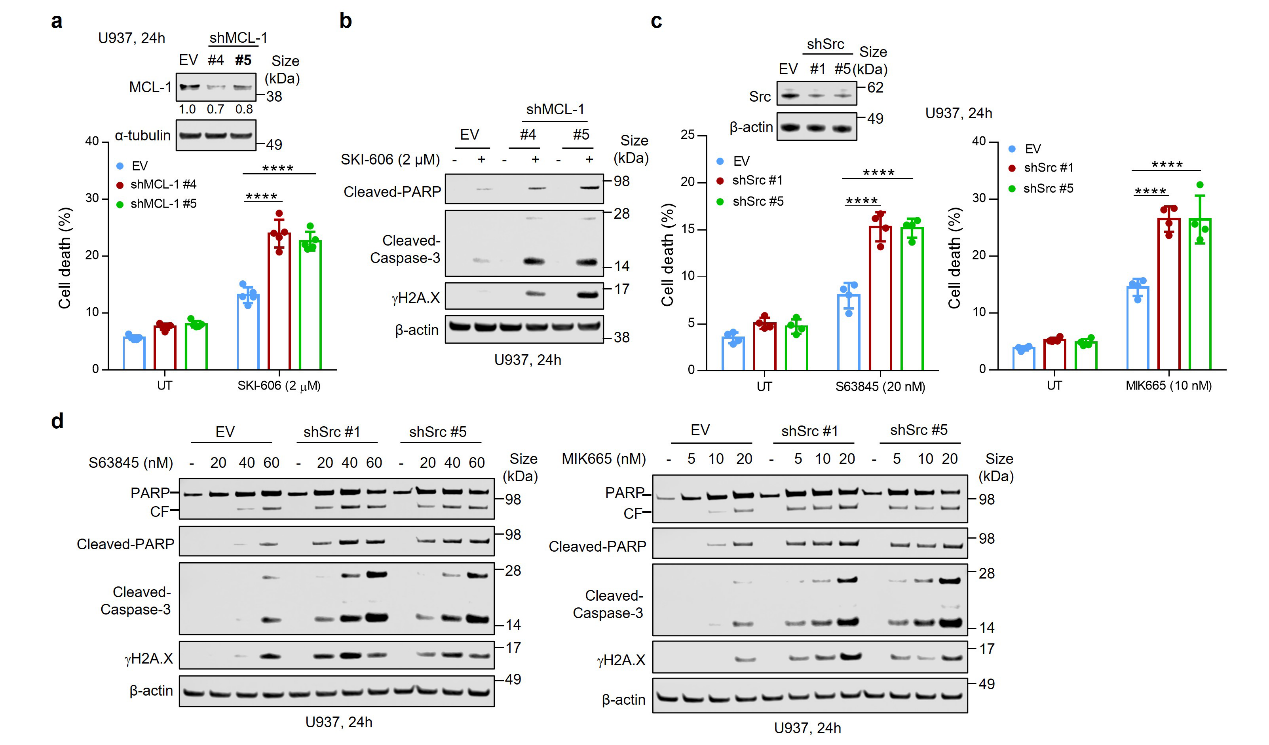


**Supplementary Figure S3.**

**MCL-1 knockdown significantly enhances the lethality of SKI-606, while Src knockdown significantly enhances the activity of MCL-1 inhibitors.**

U937 cells were infected with a lentivirus harboring MCL-1 shRNA or Src shRNA.

**a,** U937/EV and U937/shMCL-1 cells were exposed to SKI-606 (2μM) for 24 hours, after which cell death was determined by 7-AAD staining and flow cytometry (FCM) (n=5 in each group). Values represent the mean % ± SD. Inset: Expression of MCL-1 by immunoblotting after infection with a lentivirus carrying EV or shMCL-1. Numerals under the blots represent densitometric values normalized to empty vector control (1.0). *****P* < 0.0001.

**b,** Western blot analysis was performed to monitor levels of cleaved-PARP, cleaved-Caspase-3, as well as γH2A.X. β-actin was used as a loading control to ensure equal protein loading and transfer.

**c-d,** U937/EV and U937/shSrc cells were exposed to the indicated concentrations of S63845 or MIK665 for 24 hours. Assays were performed as in **a-b**. **c**, n=4 in each group. Inset: Expression of Src by immunoblotting after infection with a lentivirus carrying EV or shSrc.

EV, empty vector; CF, cleavage fragment. *****P* < 0.0001.

**Figure S4.**

**
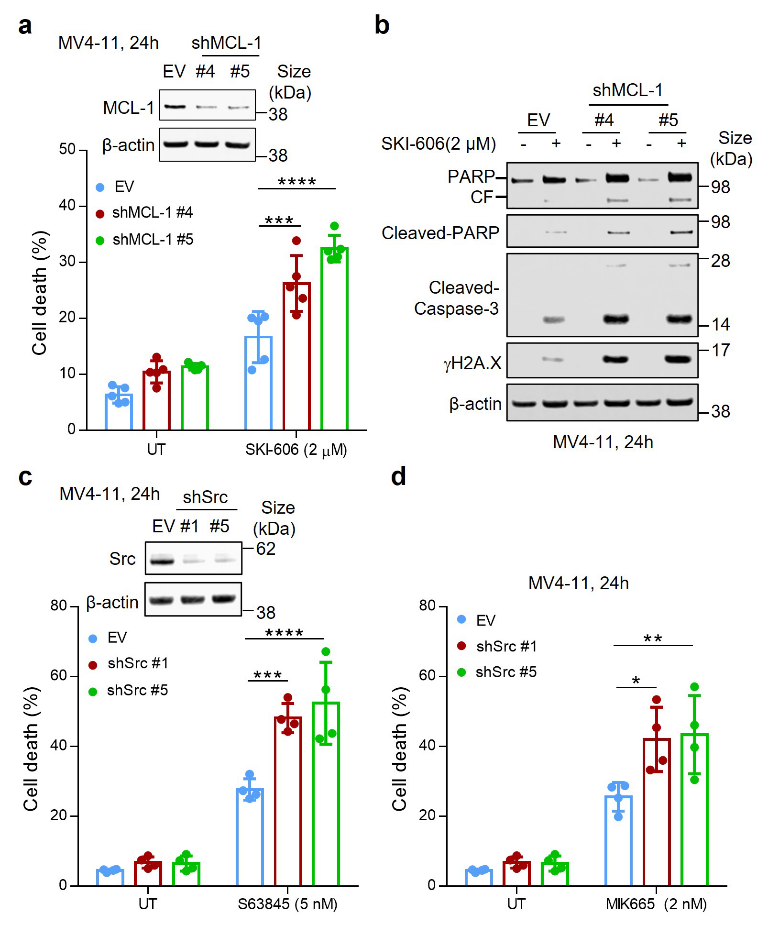
**

**Supplementary Figure S4.**

**MCL-1 knockdown significantly enhances SKI-606 lethality, while Src knockdown significantly potentiates the activity of MCL-1 inhibitors in MV4-11 cells.**

MV4-11 cells were infected with a lentivirus harboring MCL-1 shRNA or Src shRNA.

**a,** MV4-11/EV and MV4-11/shMCL-1 cells were exposed to SKI-606 (2μM) for 24 hours, after which cell death was determined by 7-AAD staining and flow cytometry (FCM) (n=5 in each group). Values represent the mean % ± SD. Inset: Expression of MCL-1 by WB after infection with a lentivirus carrying EV or shMCL-1.

**b,** Immunoblotting analysis was performed to monitor levels of PARP, cleaved-PARP and cleaved-Caspase-3, as well as γH2AX. β-actin was used as a loading control to ensure equal protein loading and transfer.

**c,** MV4-11/EV and MV4-11/shSrc cells were incubated with 5nM S63845 or 2nM MIK665 for 24 hours. Cell death was determined by 7-AAD staining and flow cytometry (FCM) (n=4 in each group). Values represent the mean % ± SD. Inset: Expression of Src by WB after infection with a lentivirus carrying EV or shSrc.

EV, empty vector; CF, cleavage fragment. **P* < 0.05, ***P* < 0.01, ****P* < 0.001, *****P* < 0.0001.

**Figure S5**

**
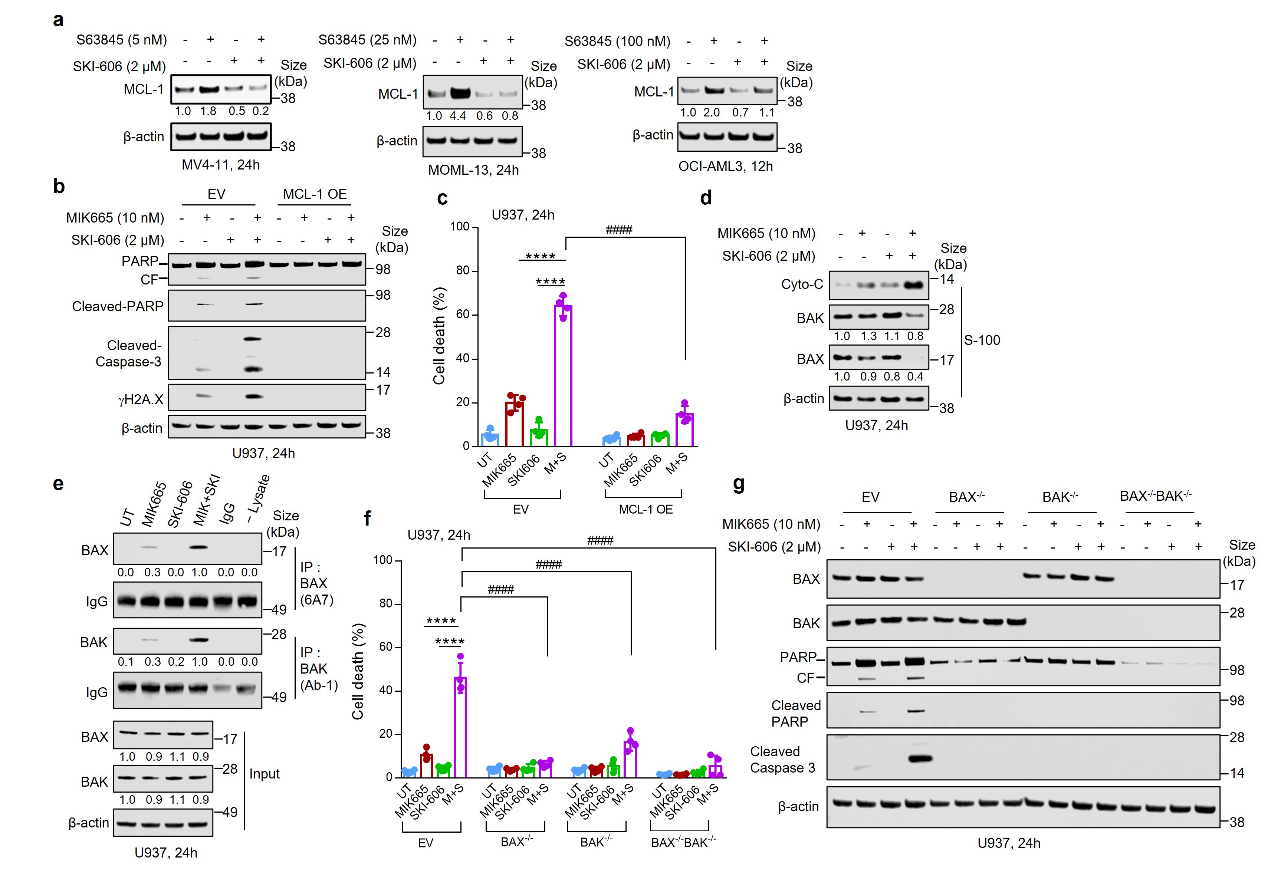
**

**Supplementary Figure S5.**

**MCL-1 down-regulation and activation of BAX and BAK are required for MCL-1 inhibitor/SKI-606-mediated cell death.**

**a,** MV4-11, MOLM-13 and AML-3 cells were exposed to the indicated concentrations of S63845 ± SKI-606 for 24 hours. Immunoblotting analysis was then performed to monitor the level of MCL-1. Numerals under the blots correspond to densitometric readings normalized to untreated controls (1.0).

**b,** U937 MCL-1 overexpressing cells were treated (24 hours) with 10nM MIK665 ± 2µM SKI-606, after which western blot analysis of PARP, cleaved-PARP and cleaved-Caspase-3, as well as γH2A.X was performed. β-actin was assayed to ensure equivalent loading and transfer.

**c,** Cell death was determined by 7-AAD staining and flow cytometric analysis. Values represent the mean % ± SD for four separate experiments.

**d,** U937 cells were exposed to MIK665 (10nM) and SKI-606 (2μM) alone or in combination for 24 hours, after which subcellular fractions were obtained and subjected to western blot analysis to monitor the release of cytochrome c, BAK, and BAX into the cytosol. S-100, cytosol; Cyto c = cytochrome c.

**e,** U937 cells were exposed to MIK665 ± SKI-606 for 24 hours after which cells were lysed in buffer containing 1% CHAPS; conformationaly changed BAX and BAK proteins were immunoprecipitated using anti-BAX 6A7 and anti-BAK Ab1 Abs, respectively, and subjected to western blot analysis using polyclonal BAX or BAK Abs.

**f,** BAK and/or BAX CRISPR knockout U937 cells were incubated with MIK665 ± SKI-606 for 24 hours. Cell death was determined using 7-AAD staining and flow cytometry (n=4 in each group). Values represent the mean % ± SD.

**g,** BAK, BAX, PARP, cleaved-PARP and cleaved-Caspase-3, as well as γH2A.X were detected by western blot. β-actin was assayed to ensure equivalent loading and transfer.

CF, cleavage fragment; EV, empty vector; OE, overexpression. *****P* < 0.0001; ^####^*P* < 0.0001.

**Figure S6**


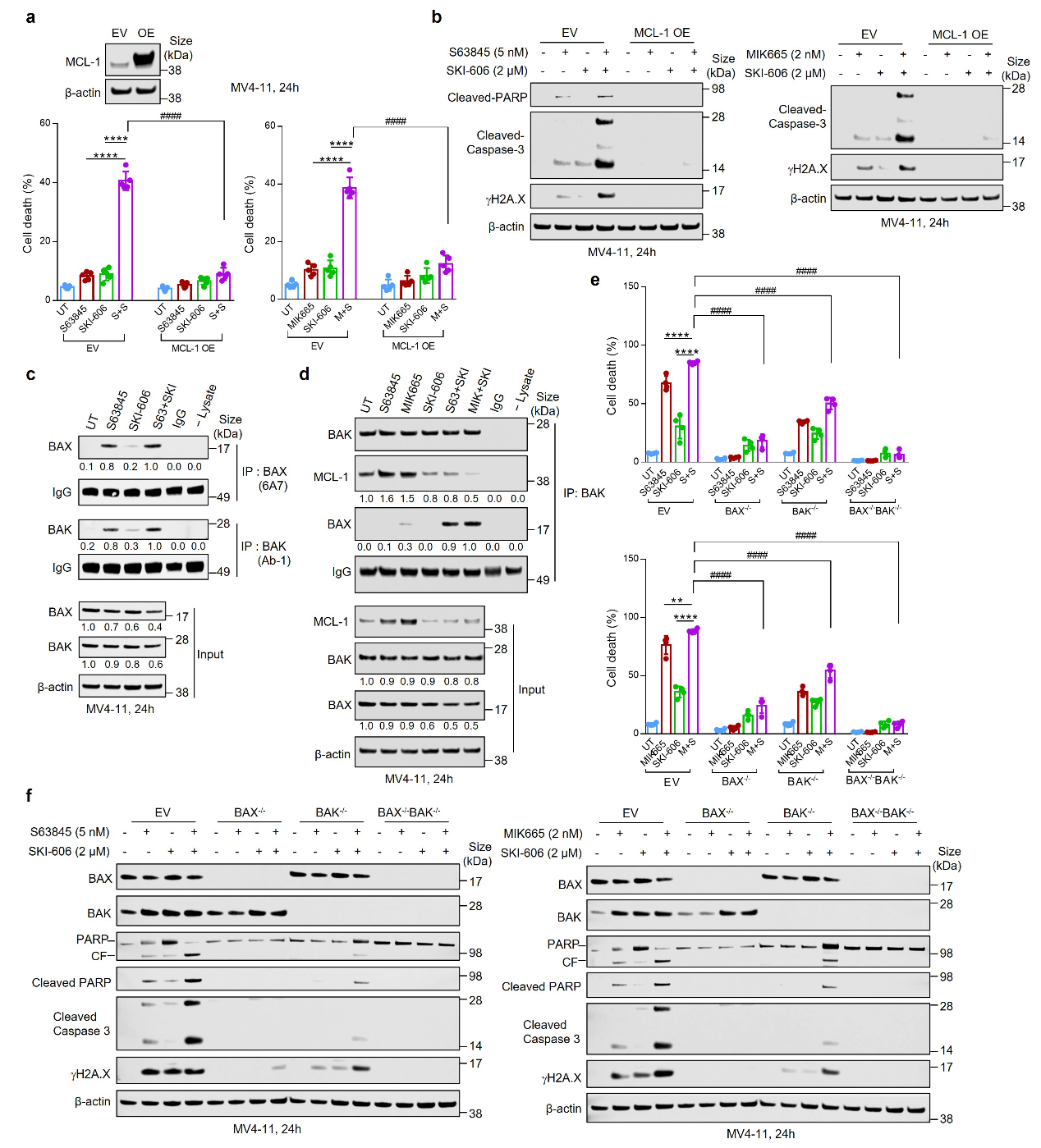


**Supplementary Figure S6.**

**MCL-1 down-regulation and activation of BAX and BAK are required for MCL-1 inhibitor/SKI-606-mediated cell death in MV4-11 cells.**

**a-b,** Ectopic expression of MCL-1 in MV4-11 cells.

**a,** MV4-11/EV or MV4-11/MCL-1 OE cells were treated (24 hours) with 5nM S63845 or 2nM MIK665 ± 2µM SKI-606, after which cell death was determined by 7-AAD staining and flow cytometry (n=5 in each group). Values represent the mean % ± SD.

**b,** Cleaved-PARP and Cleaved-Caspase-3, as well as γH2A.X were detected by western blot analysis. β-actin was assayed to ensure equivalent loading and transfer.

**c,** MV4-11 cells were exposed to 5nM S63845 ± 2µM SKI-606 for 24 hours, after which cells were lysed in buffer containing 1% CHAPS; conformationaly changed BAX and BAK proteins were immunoprecipitated using anti-BAX 6A7 and anti-BAK Ab1 Abs, respectively, and subjected to western blot analysis using polyclonal BAX or BAK Abs. Numerals under the blots correspond to densitometric readings normalized to untreated controls (1.0).

**d,** Following 24 hours treatment, MV4-11 cells were lysed in buffer and immunoprecipitated (IP) using anti-BAK antibody, followed by western blot analysis using anti-BAK, anti-BAX, or anti-MCL-1 antibodies as indicated. For all IP assay, IPs without cell lysate (-lysate) and/or with IgG (instead of primary antibodies) were carried out as controls; input lysates were also subjected to western blot analysis to monitor relative protein levels. IgG levels are shown to ensure equal loading of IP antibodies.

**e,** CRISPR BAK and/or BAX knockout MV4-11 cells were treated with 5nM S63845 or 2nM MIK665 ± 2μM SKI-606 for 24 hours, after which cell death was determined by 7-AAD staining and flow cytometry (n=4 in each group). Values represent the mean % ± SD.

**f,** BAK, BAX, PARP, cleaved-PARP, cleaved-Caspase-3 and γH2A.X were detected by western blot analysis. β-actin was assayed to ensure equivalent loading and transfer.

EV, empty vector; OE, overexpression; CF, cleavage fragment. ***P* < 0.01, ****P* < 0.001, *****P* < 0.0001, ^##^*P* <0.01, ^####^*P* <0.0001.

**Figure S7**


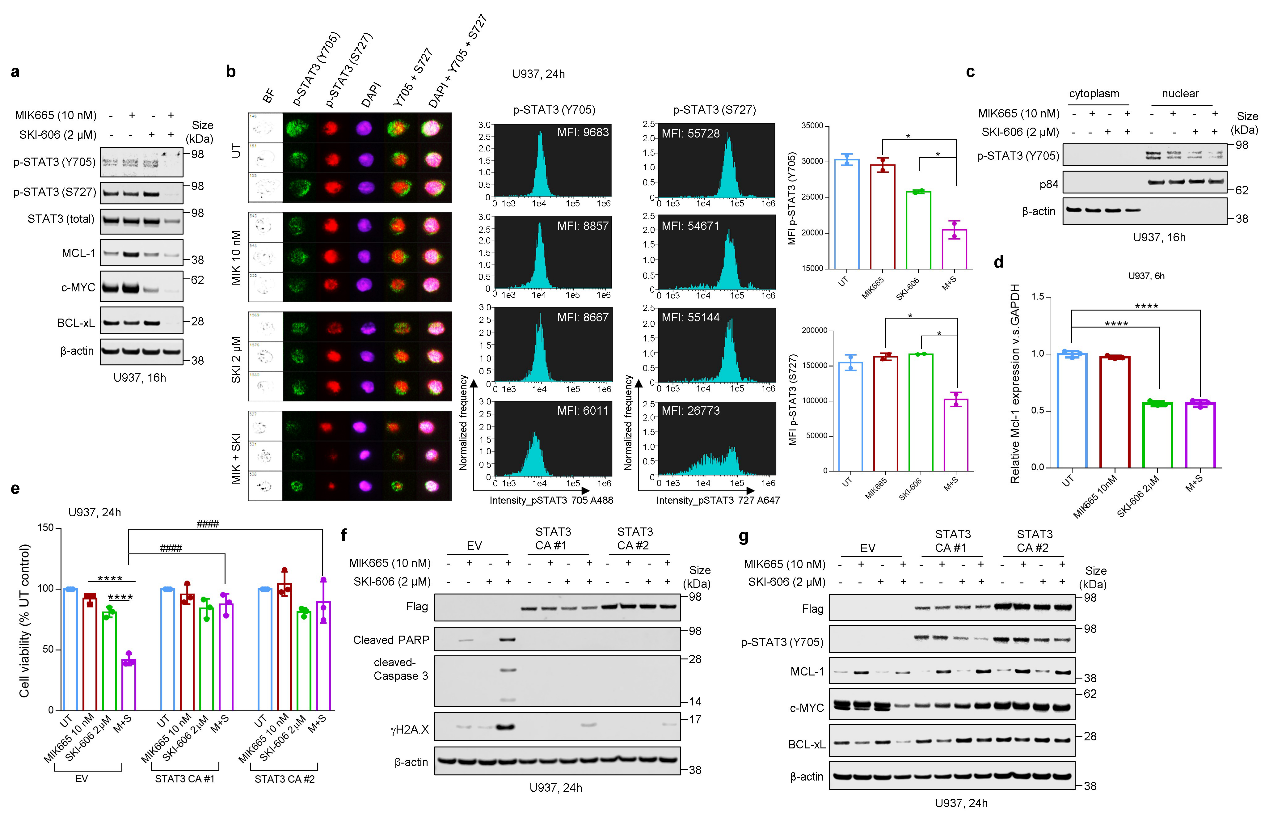


**Supplementary Figure S7.**

**The MIK665/SKI-606 regimen blocks phosphorylation of STAT3 (p-Y705 and p-S727) and diminishes MCL-1 transcription by reducing p-STAT3 (Y705) nuclear translocation.**

**a,** Western blot analysis of p-STAT3 (Y705), p-STAT3 (S727), MCL-1, c-MYC, BCL-_X_L in U937 cells treated with 10nM MIK665 ± 2µM SKI-606 for 16 hours.

**b,** Following 24 hours treatment, cells were stained with p-STAT3 (Y705), p-STAT3 (Y727) and DAPI, and then visualized by fluorescence microscope and by ImageStream; representative cells are shown (BF = brightfield). Histograms of p-STAT3 (Y705) and p-STAT3 (Y727) intensity and fold change are shown (n=2 in each group). Values represent the mean ± SD.

**c,** U937 cells were treated with MIK665 ± SKI-606 for 16 hours. Nuclear and cytoplasmic extracts were collected for performance of p-STAT3 Y705 western blot assays. P84 and β-actin were used as loading controls for nuclear and cytoplasmic protein, respectively.

**d,** Relative mRNA expressions of MCL-1 was determined by real-time RT-PCR analysis (n=3 in each group). Values represent the mean ± SD. GAPDH served as an internal control.

**e,** U937 cells carrying empty vector (EV) or STAT3-CA were exposed (24 hours) to indicated concentrations of MIK665 ± SKI-606, followed by CellTiter-Glo^®^ Luminescent Cell Viability Assay to monitor cell viability. Values represent the mean % ± SD for three separate experiments performed in triplicate.

**f**, Western blot analysis of FLAG, cleaved-PARP and cleaved-Caspase-3, as well as γH2A.X was performed. β-actin were assayed to ensure equivalent loading and transfer.

**g**, Western blot analysis of FLAG, p-STAT3 (Y705), MCL-1, C-MYC, as well as BCL-xL was performed. β-actin were assayed to ensure equivalent loading and transfer.

EV, empty vector. **P* < 0.05, *****P* < 0.0001; ^####^*P* <0.0001.

**Figure S8**


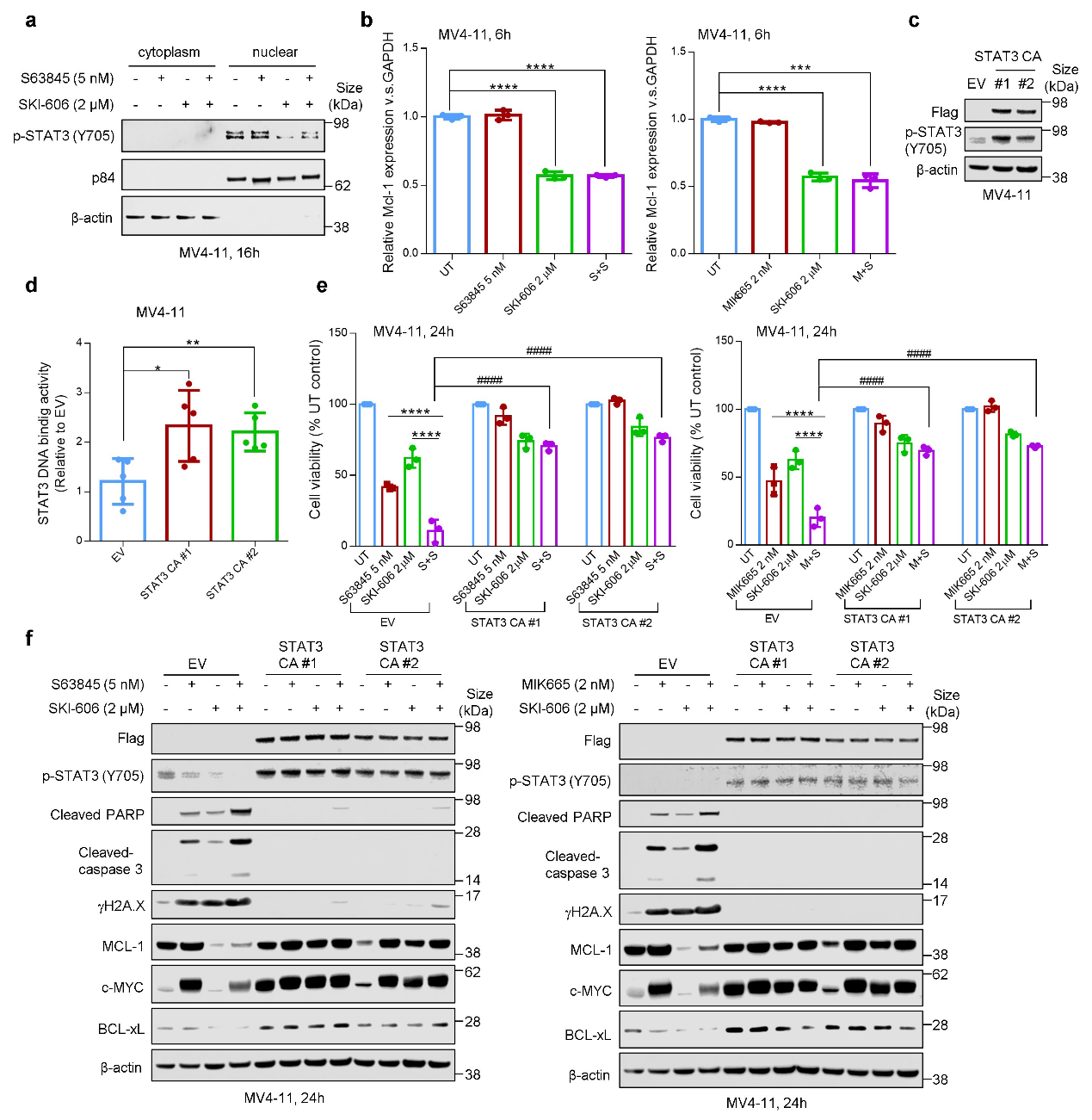


**Supplementary Figure S8.**

**MCL-1 inhibitor/SKI-606 exposure diminishes MCL-1 transcription by reducing p-STAT3 (Y705) nuclear translocation, and induces apoptosis in a STAT3-dependent manner** **in MV4-11 cells.**

**a,** MV4-11 cells were treated with 5nM S63845 ± 2μM SKI-606for 16 hours, after which nuclear and cytoplasmic extracts were collected for performance of p-STAT3 Y705 western blot assays. P84 and β-actin were used as loading controls for nuclear and cytoplasmic protein, respectively.

**b,** Relative mRNA expressions of MCL-1 was determined by real-time RT-PCR analysis (n=3 in each group). Values represent the mean ± SD. GAPDH served as an internal control.

**c-f,** MV4-11 cells were infected with a lentivirus harboring constitutively-active STAT3 (FLAG fusion), and clones CA-STAT3 #1 and CA-STAT3 #2 cells were selected for subsequent experiments.

**c,** Western blot was performed to test Flag and p-STAT3 (Y705) level in CA-STAT3 MV4-11 cells.

**d,** The STAT3 DNA-binding ELISA assay was used to evaluate STAT3 activity in CA-STAT3 MV4-11 cells (n=5 in each group). Values represent the mean ± SD.

**e,** MV4-11 cells carrying EV or CA-STAT3 were exposed (24 hours) to indicated concentrations of S63845 or MIK665 ± SKI-606, followed by CellTiter-Glo^®^ Luminescent Cell Viability Assay to monitor cell viability. Values represent the means % ± SD for three separate experiments performed in triplicate.

**f,** Western blot analysis of FLAG, p-STAT3 (Y705), cleaved-PARP, cleaved-Caspase-3, γH2A.X, MCL-1, C-MYC, as well as BCL-xL was performed. β-actin were assayed to ensure equivalent loading and transfer.

EV, empty vector. **P* < 0.05, ***P* < 0.01, ****P* < 0.001, *****P* < 0.0001; ^####^*P* < 0.0001.

**Figure S9**


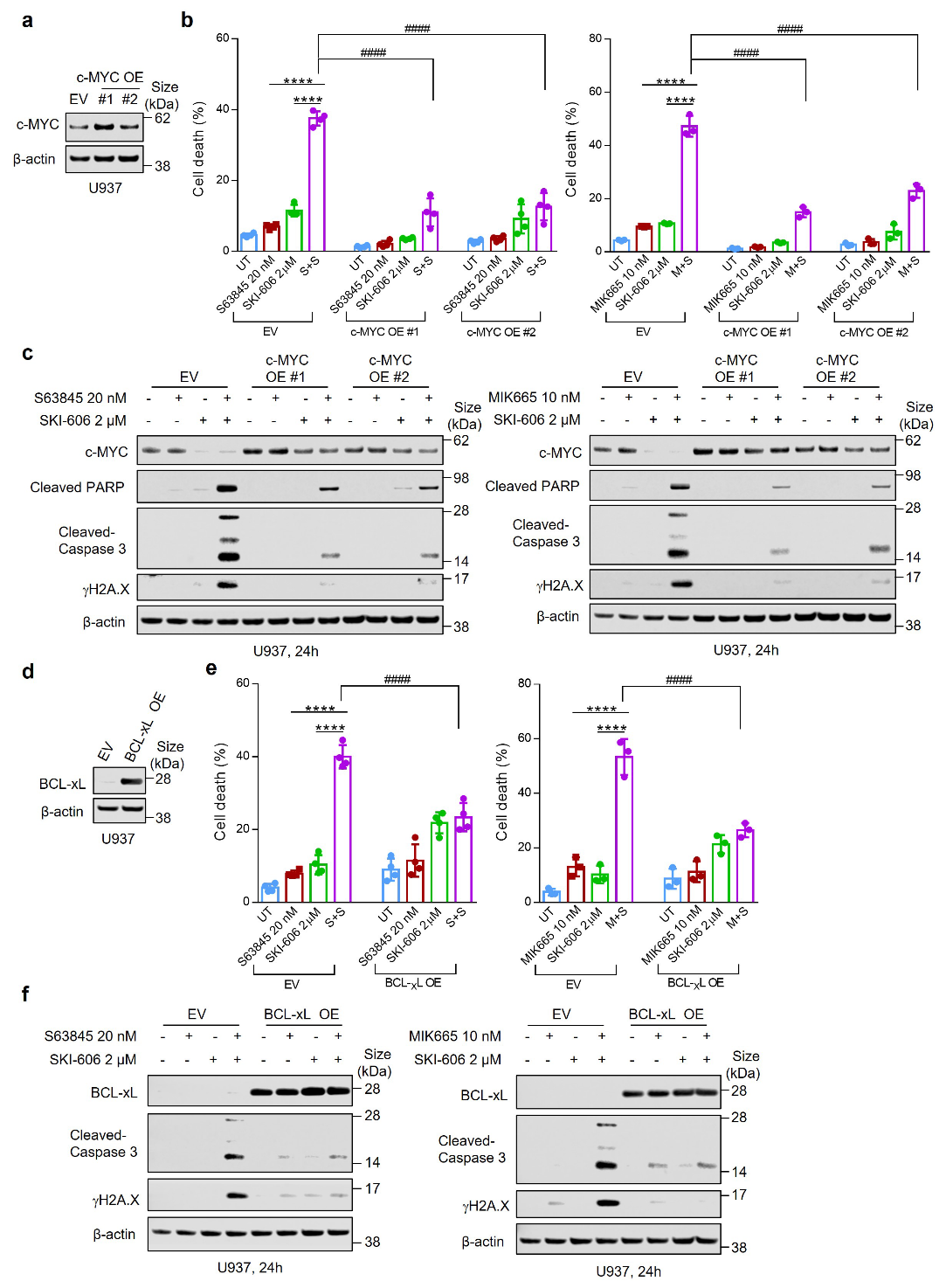


**Supplementary Figure S9.**

**Overexpression of c-MYC or BCL-_X_L diminishes cell death induced by co-treatment with MCL-1 inhibitors and SKI-606 in U937 cells.**

**a-c,** U937 cells were transfected with pMYC-GFP construct (c-MYC overexpression), clone #1 and clone #2 were selected for the subsequent experiments.

**a,** Western blotting analysis was performed to assess c-MYC levels.

**b,** Cells carrying either EV or exhibiting c-Myc over-expression were exposed (24 hours) to indicated concentrations of S63845/MIK665 ± SKI-606, followed by flow cytometric analysis of cell death after staining with 7-AAD. Values represent the means % ± SD for at least three separate experiments performed in triplicate.

**c,** Western blot analysis of c-MYC, cleaved-PARP, cleaved-Caspase-3, and γH2A.X was performed. β-actin were used to ensure equal loading and transfer.

**d-f,** U937 cells were transfected with either EV or pCDH-puro-BCL-xL construct. Assays were performed as in **a-c**. **e**, Values represent the means % ± SD for at least three separate experiments performed in triplicate.

EV, empty vector; OE, overexpression. *****P* < 0.0001; ^####^*P* < 0.0001.

**Figure S10**


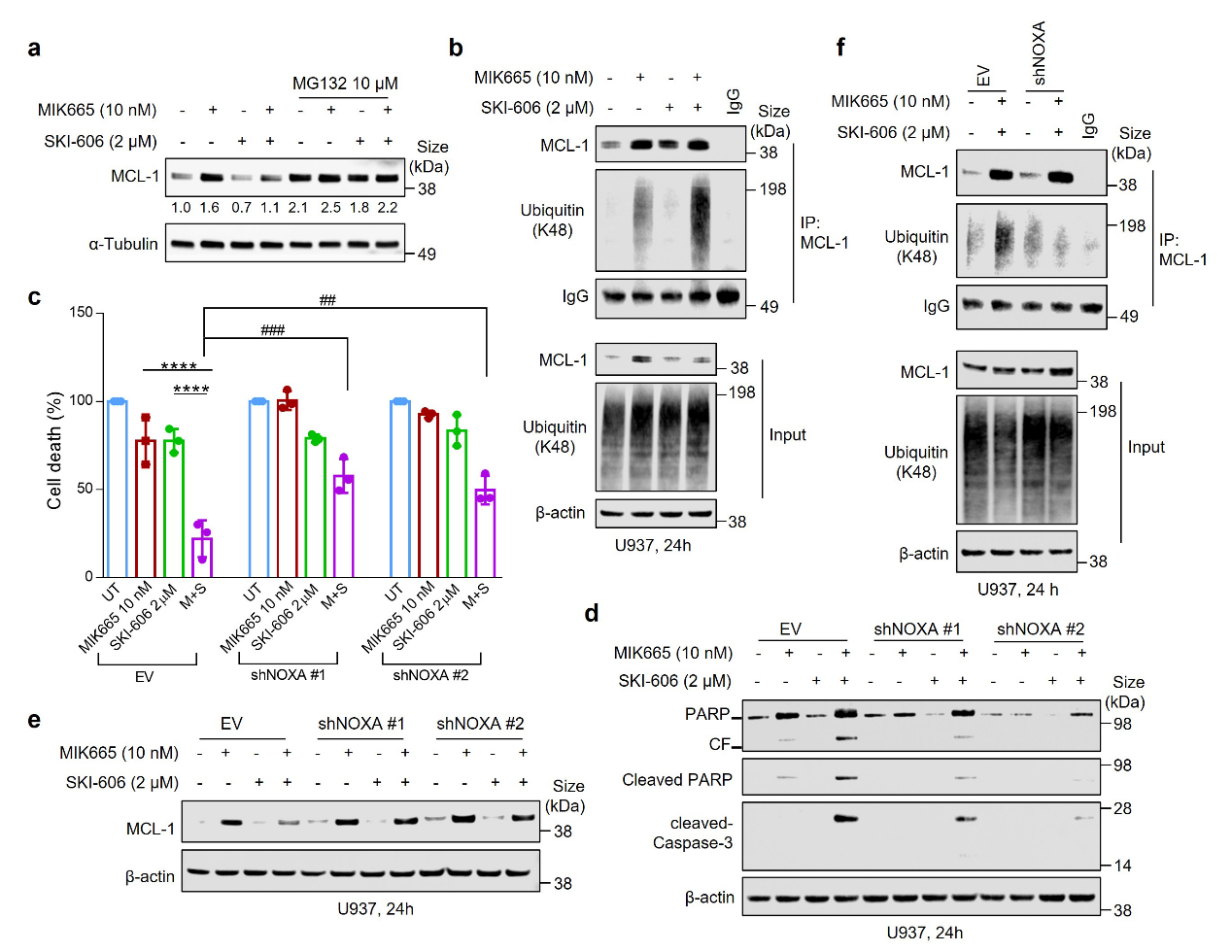


**Supplementary Figure S10.**

**Co-treatment with MIK665 and SKI-606 promotes NOXA and ubiquitination-related degradation of MCL-1 in U937 cells.**

**a,** Effect of MG132 on MCL-1 expression in U937 cells treated with MIK665 ± SKI-606. The cells were pretreated with 10nM MIK665 ± 2µM SKI-606 for 16 hours, then incubated with 10 µM MG132 for 2 hours. Numerals under the blots correspond to densitometric readings normalized to untreated controls (1.0).

**b,** U937 cells were exposed to the indicated concentrations of MIK665 ± SKI-606 for 24 hours, after which cells were lysed in buffer and immunoprecipitated (IP) using anti-MCL-1 antibody, followed by western blot analysis using anti-ubiquitin K48 antibody as indicated; input lysates were also subjected to western blot analysis to monitor relative protein levels. IgG levels are shown to ensure equal loading of IP antibodies.

**c,** U937 cells carrying empty vector (EV) or shNOXA were exposed to 10nM MIK665 ± 2μM SKI-606 for 24 hours, after which cell viability was determined by CellTiter-Glo^®^ Luminescent Cell Viability Assay. Values represent the means % ± SD for three experiments performed in triplicate. *****P* < 0.0001; ^##^*P* < 0.01, ^####^*P* < 0.0001.

**d,** Western blot analysis of PARP, cleaved-PARP and cleaved-Caspase-3 was performed. β-actin controls were assayed to ensure equivalent loading and transfer. CF, cleavage fragment.

**e,** Effect of NOXA depletion on MCL1 expression.

**f**, Ubiquitination levels of MCL-1 in U937 NOXA knock down cells treated with MIK/SKI.  IP with IgG (instead of primary antibodies) were carried out as controls.

**Figure S11**

**
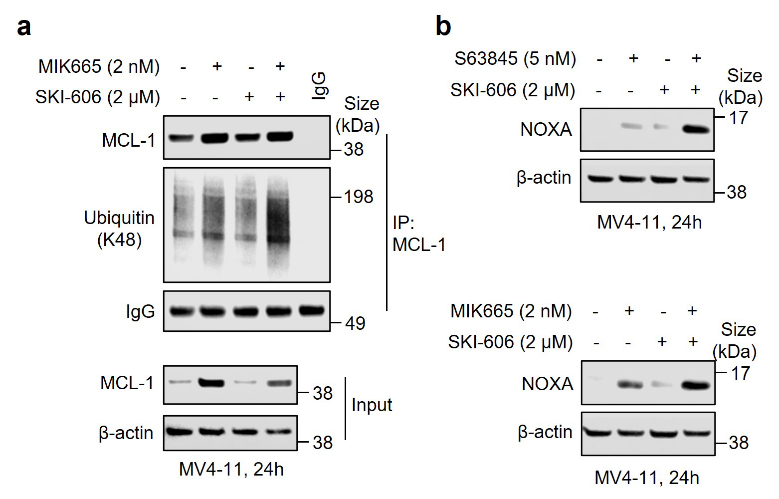
**

**Supplementary Figure S11.**

**Co-treatment with MCL-1 inhibitors and SKI-606 promotes NOXA and ubiquitination-related degradation of MCL-1 in MV4-11 cells.**

**a,** MV4-11 cells were exposed to the indicated concentrations of MIK665 ± SKI-606 for 24 hours, after which cells were lysed in buffer and immunoprecipitated (IP) using anti-MCL-1 antibody, followed by western blot analysis using anti-ubiquitin K48 antibody as indicated; input lysates were also subjected to western blot analysis to monitor relative protein levels. IgG levels are shown to ensure equal loading of IP antibodies.

**b,** Western blot analysis showing the expression of NOXA in MV4-11 cells after treatment with S63845/MIK665 ± SKI-606 (24 hours).

**Figure S12**


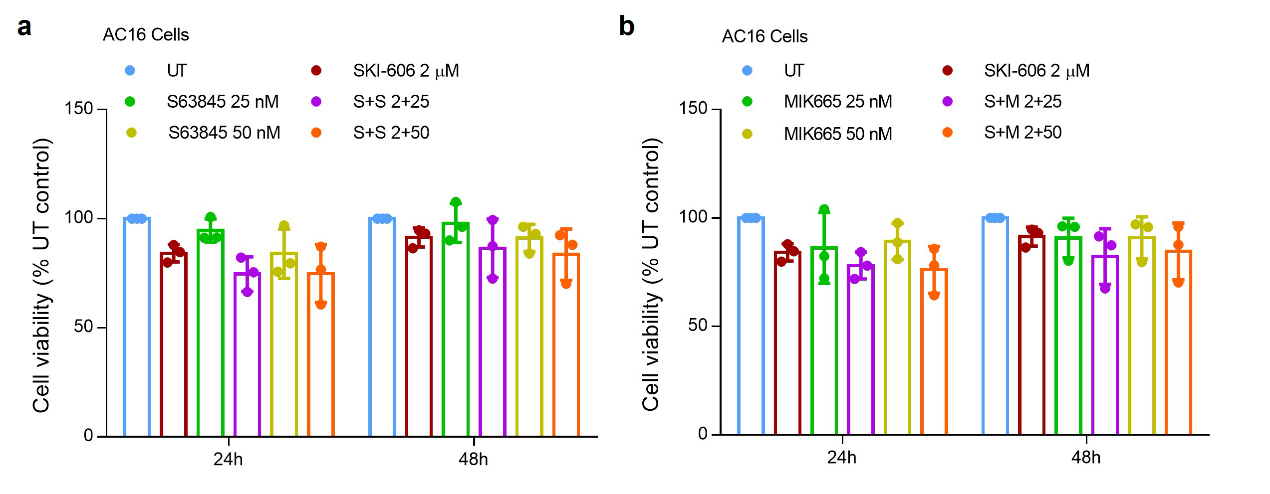


**Supplementary Figure S12.**

**The MCL-1/Src antagonist regimen is non-toxic toward normal human adult ventricular cardiomyocyte cells (AC16).**

**a** and **b,** AC16 adult ventricular cardiomyocyte cells were exposed (24 hours and 48 hours) to the indicated concentrations of S63845 / MIK665 ± SKI-606, followed analysis by the CellTiter-Glo® Luminescent Cell Viability Assay to monitor cell viability. Values represent the means % ± SD for three separate experiments performed in triplicate (*P* > 0.05).

**Figure S13**


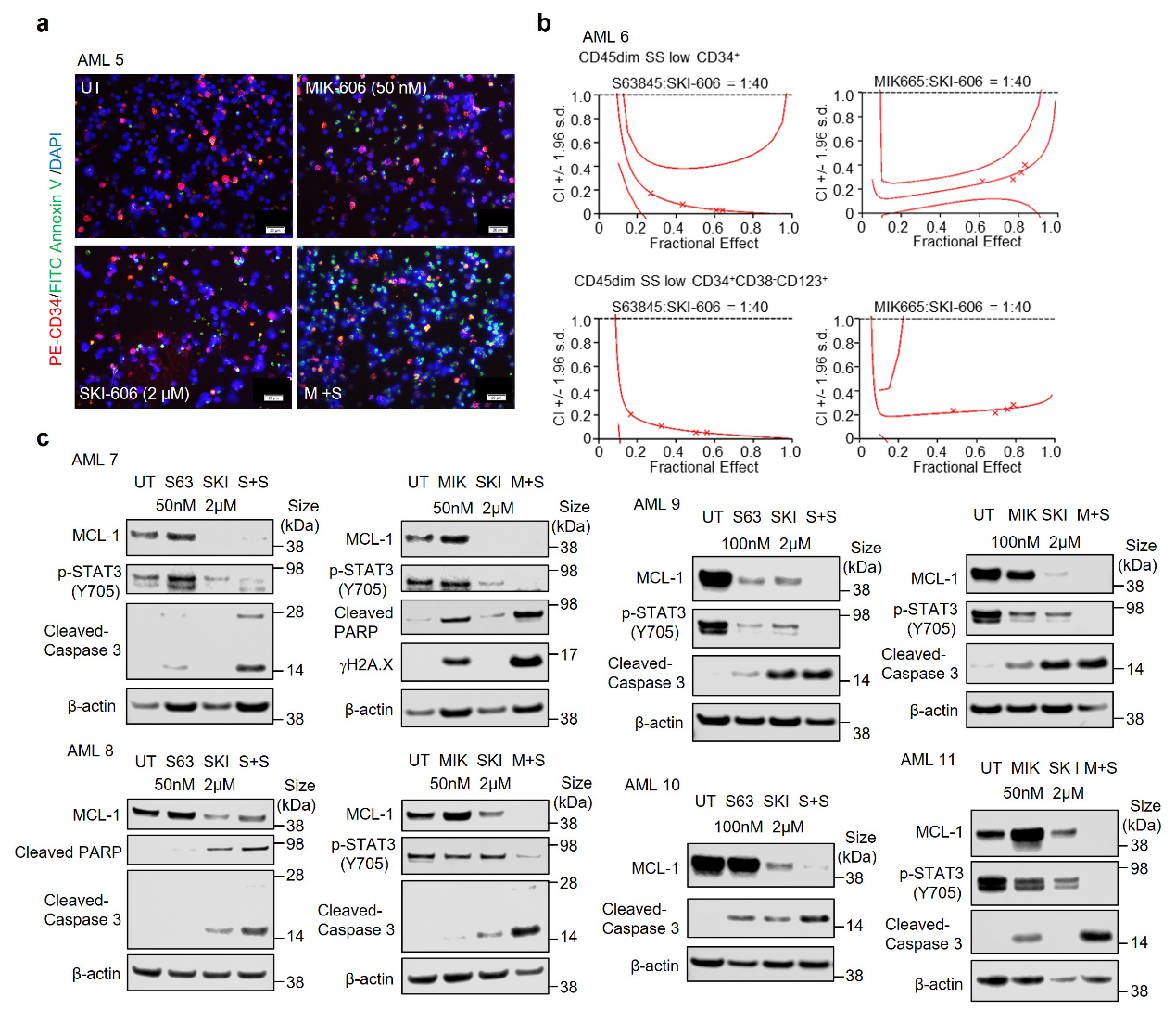


**Supplementary Figure S13.**

**MCL-1 inhibitors/SKI-606 exposure kills primary AML blasts including CD34^+^CD123^+^CD38^-^ progenitors while blocking STAT3 phosphorylation (p-Y705) and triggering MCL-1 down-regulation.**

**a,** Representative primary bone marrow cells from a patient with AML were exposed to 50nM MIK665 ± 2µM SKI-606 for 24 hours, after which the cells were stained with PE-CD34, FITC-annexin V and DAPI. Scale bar = 20µm.

**b,** Primary AML patient samples were exposed (16~20 hours) to varying concentrations of S63845 / MIK665 ± SKI-606 at a fixed ratio (1:40), after which the percentage of annexin V^+^ cells was determined. Median dose-effect analysis was then employed to characterize the nature of the interaction between these agents. Combination index values <1.0 denote a synergistic interaction.

**c,** Western blot analysis of MCL-1, p-STAT3 Y705, cleaved-PARP, cleaved-Caspase-3, as well as γH2A.X in representative primary patient samples following exposure to S63845/MIK665 50nM or 100nM ± SKI-606 2µM for 16~20 hours. β-actin controls were assayed to ensure equivalent loading and transfer.

**Figure S14**


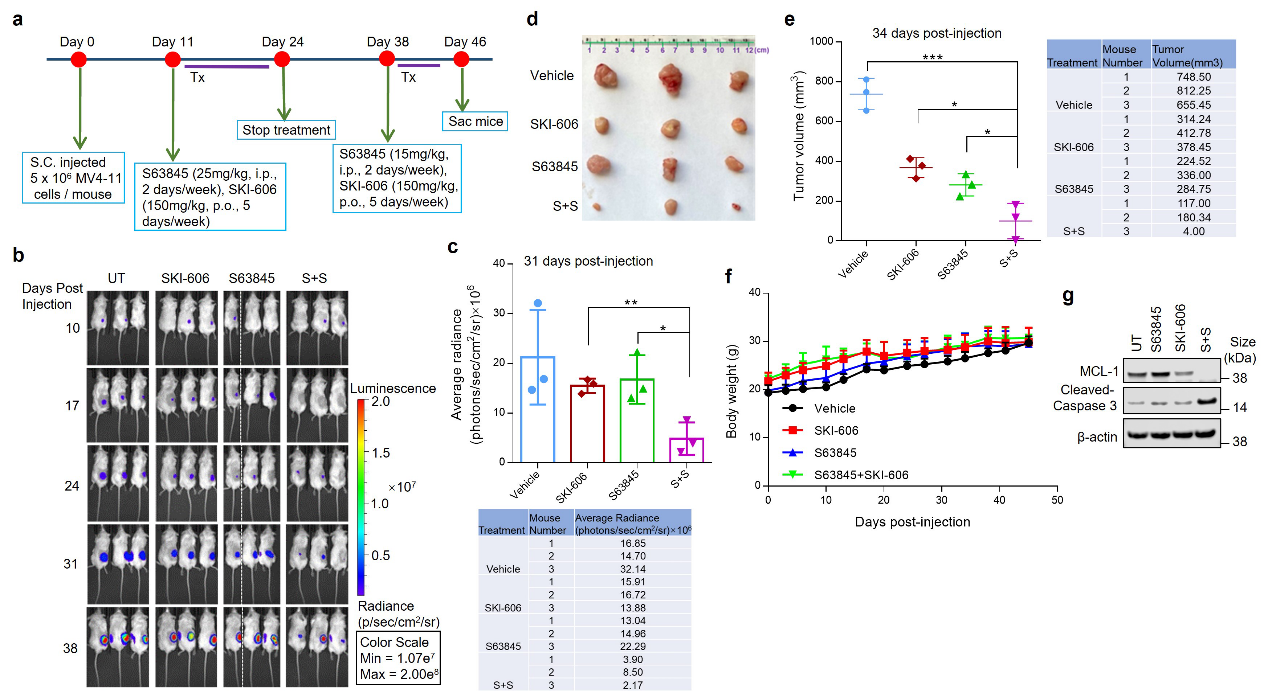


**Supplementary Figure S14.**

**Co-administration of S63845 and SKI-606 suppresses tumor growth in an MV4-11 cell xenograft model.**

NOD/SCID-γ (NSG) mice (3 mice/group) were inoculated in the right flank with 5 x 10^6^ MV4-11-Luc cells. Treatment was initiated after 11 days. S63845 (25mg/kg, twice a week, I.P.) ± SKI-606 (150mg/kg, 5 days weekly, p.o.) were administrated per week. Body weight and tumor size were monitored every other day. Mice were euthanized when tumor size reached 17 mm (length) or other humane endpoints (e.g., abscessed or necrotic tumors) were reached.

**a**, Timeline of cell inoculation, administration of S63845 and SKI-606, and sacrifice of mice.

**b,** Tumor burden was monitored every week after sub-Q injection with 150 mg/kg luciferin using the IVIS 200 imaging system.

**c,** Quantification of the luminescent signal at day 31. Data represents the mean ± SD performed on all mice for each group. **P*<0.05, ***P*<0.01.

**d,** Tumor images of MV4-11 cell xenografts.

**e**, Tumor sizes at day 34. **P* <0.05, ****P* <0.001.

**f**, Body weights were monitored every other day. Data are shown as the mean ± SD. *P* >0.05.

**g**, Immunoblotting analysis was then performed to monitor levels of proteins shown in the figure. β-actin controls were assayed to ensure equivalent loading and transfer.

**Figure S15**


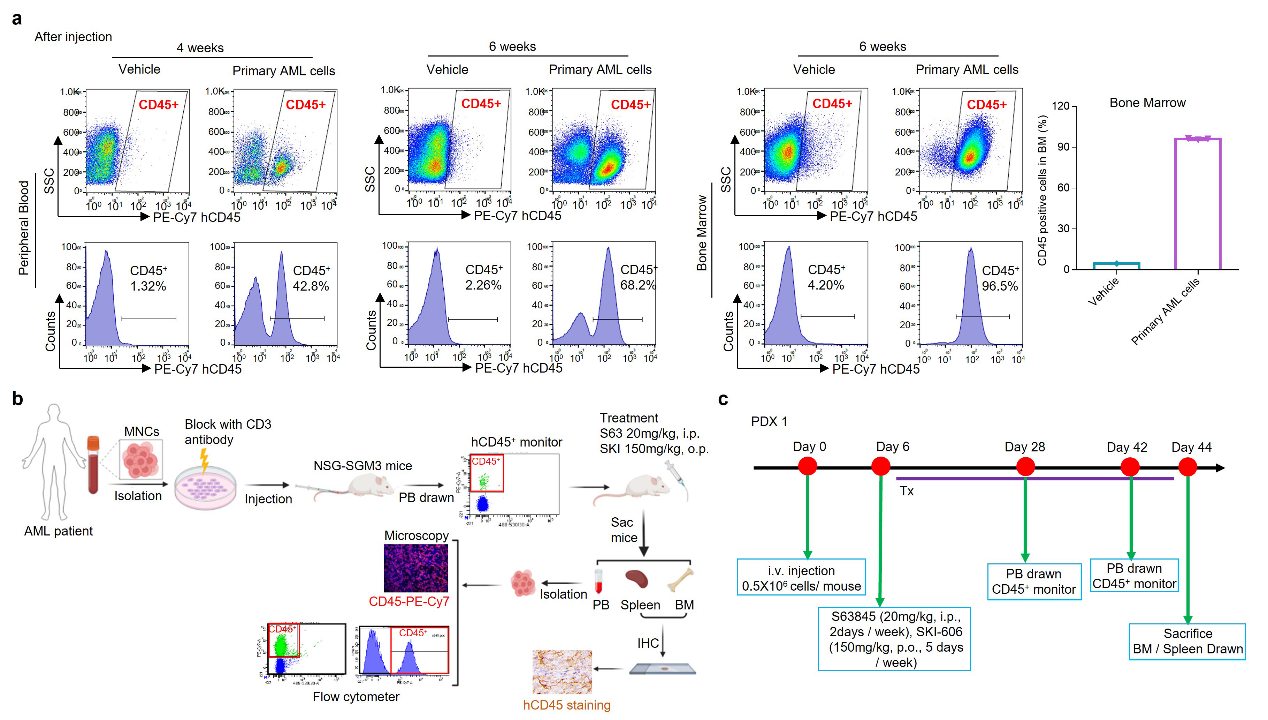


**Supplementary Figure S15.**

**a,** Analysis of human CD45^+^ (hCD45^+^) cells for patient-derived primary AML specimens (#01) used in the PDX model. NOD/SCID-gamma SCF/GM-CSF/IL3 (NSG-SGM3) mice (n=3) were inoculated via tail vein with 5 ×10^6^ primary patient AML cells. After 4 and 6 weeks, hCD45^+^ cells in the peripheral blood and bone marrow were quantified by flow cytometry.

**b,** Schematic outline illustrating the establishment of a PDX model and subsequent *in vivo* assays.

**c,** Timeline of cell inoculation, peripheral blood monitoring, administration of S63845 and SKI-606, and sacrifice of animals (PDX model 1).

**Figure S16**


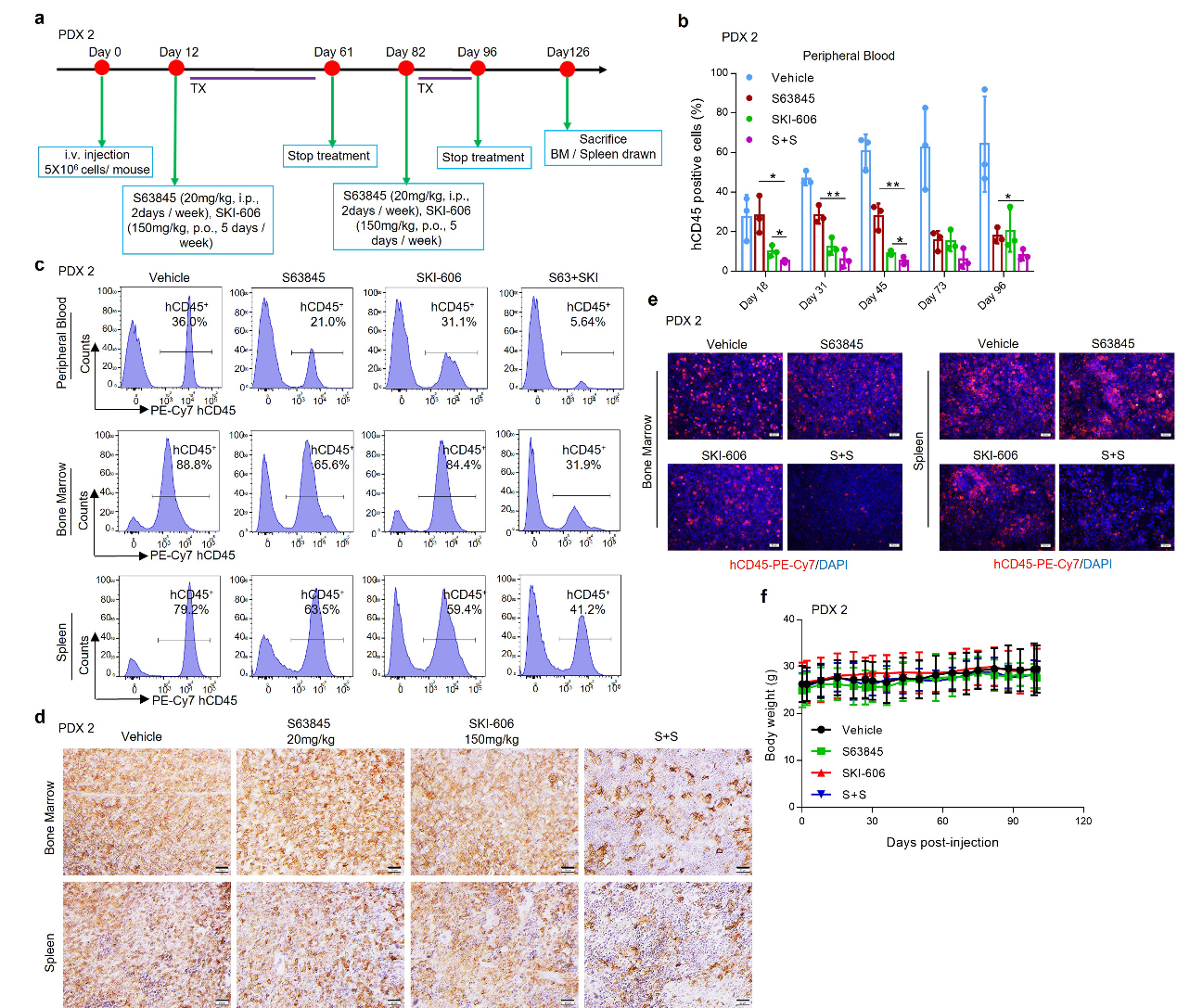


**Supplementary Figure S16.**

**Combined S63845 / SKI-606 exposure inhibits leukemic cell expansion in a second PDX model.**

NOD/SCID-gamma SCF/GM-CSF/IL3 (NSG-SGM3) mice (4 mice/group) were inoculated via tail vein with 5 ×10^6^ primary patient AML cells (#02). Treatment was initiated after 12 days. S63845 (20mg/kg, twice a week, I.P.) ± SKI-606 (150mg/kg, 5 days weekly, p.o.) were administrated weekly for 9 weeks. Control animals were administered equal volumes of vehicle. Human CD45^+^ cells in the peripheral blood were monitored every two weeks.

**a,** Timeline of cell inoculation, administration of S63845 and SKI-606, and sacrifice of animals.

**b,** hCD45^+^ cells in the peripheral blood were quantified by flow cytometry (n=3 in each group). Data are shown as the mean % ± SD. **P* < 0.05, ***P* < 0.01.

**c,** The percentage of hCD45^+^ cells in the peripheral blood, bone marrow, and spleen in different mouse groups was reflected in the histogram.

**d,** IHC of bone marrow (femur) and spleen, stained with monoclonal antibody for hCD45 in experimental mice. Scale bar = 50μm.

**e,** Representative primary bone marrow cells and spleen cells extracted from PDX mice were stained with hCD45-PE/Cy7 and DAPI to identify hCD45^+^ cells. Scale bar = 20μm.

**f,** Body weights were monitored every other day during the treatment interval. Data are shown as the mean ± SD. *P* >0.05.

**Figure S17**

**
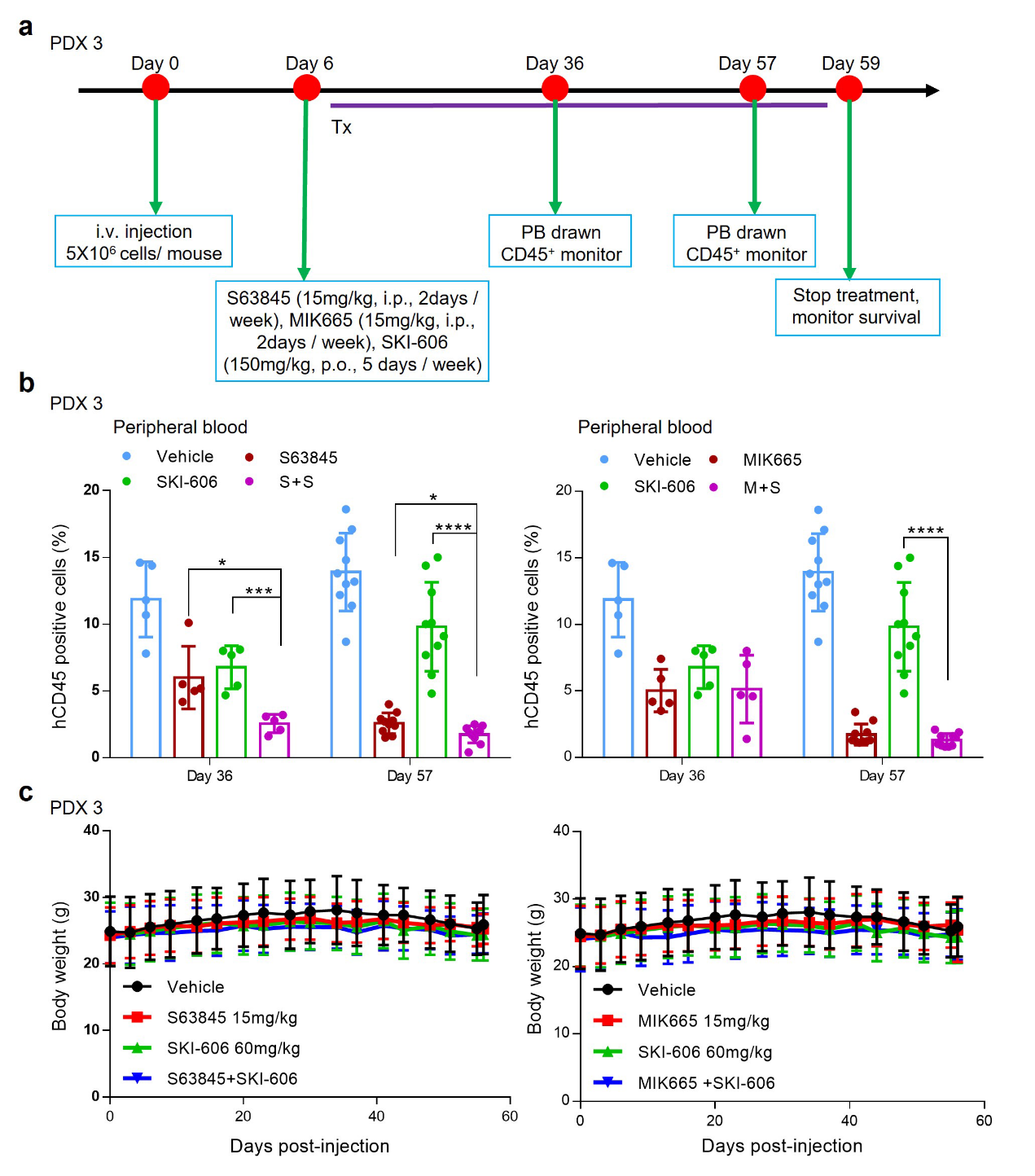
**

**Supplementary Figure S17.**

**a,** Timeline of cell inoculation, administration of drug, and peripheral blood monitoring in a third PDX survival model.

**b,** Quantification of human CD45^+^ cells in the peripheral blood by flow cytometry. At 36 days, 5 mice from each group are randomly selected for testing. At 57 days, all mice in each group (n=10) were tested. Data are shown as the mean % ± SD. **P* <0.05, ****P* <0.001, *****P* <0.001.

**c,** Mouse body weights during treatment. Data are shown as the mean ± SD. *P*>0.05.

**Figure S18.**


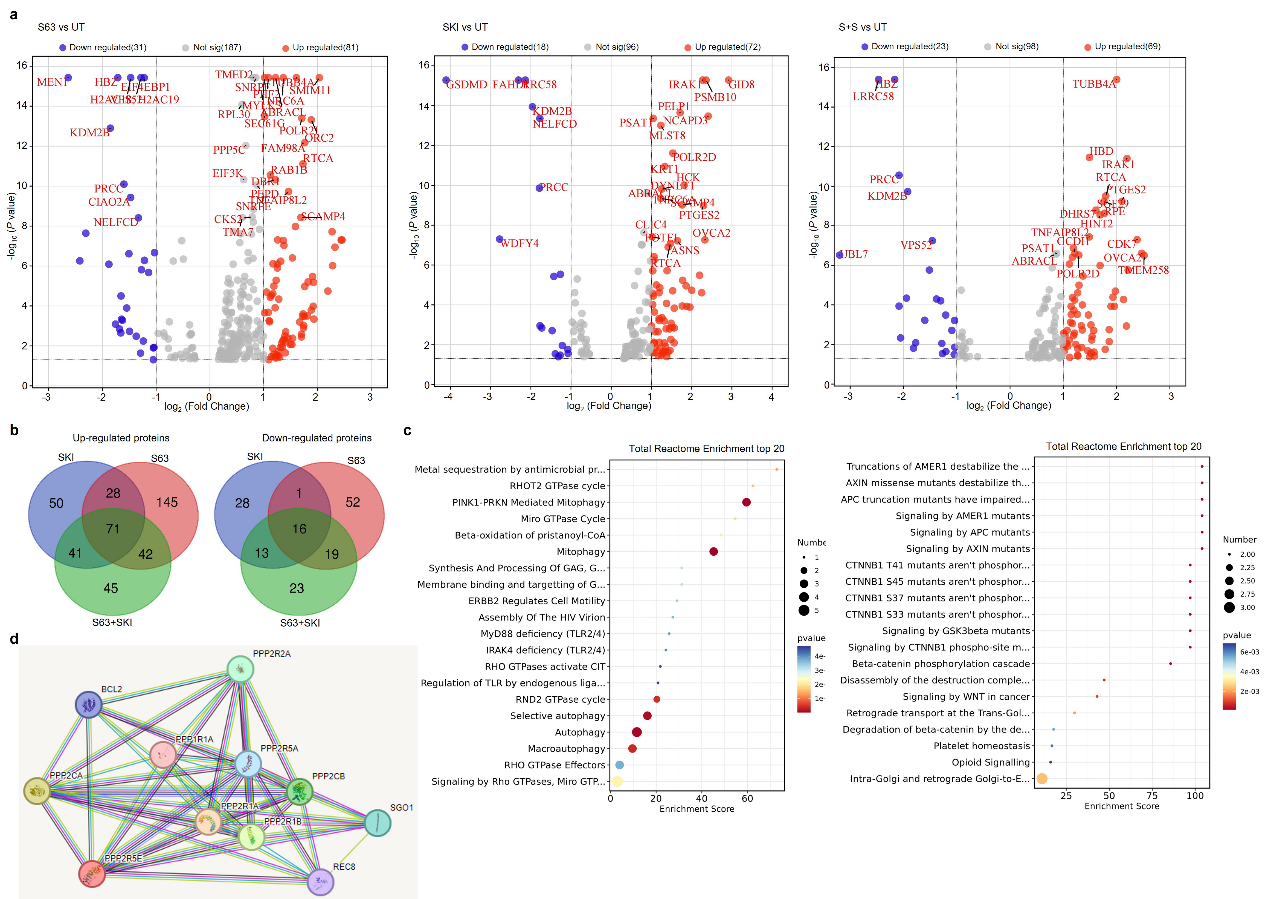


**Supplementary Figure S18.**

**Proteomics analysis.**

**a,** Volcano plot of differentially expressed proteins (DEPs) in each treatment group vs control group (UT). Red represents the protein that were significantly up-regulated, while blue indicates significantly down-regulated proteins. Gray dots represent proteins that did not show significant changes. The vertical lines mark the significant threshold filters.

**b,** Venn diagrams illustrate the number of up (left)- and down (right) -regulated proteins in each treatment group vs control group (UT).

**c,** Top 20 reactome pathway enrichment analyses of 45 up-regulated(left) and 23 down-regulated (right) protein only found in the combined treatment group (S63+SKI). The x-axis demonstrates enrichment score. The color of each point indicates the significance of the enrichment (with red representing strong significance); the size of each point corresponds to the number of proteins associated with the respective pathway.

**d,** STRING protein-protein interaction network.

**Figure S19.**

**
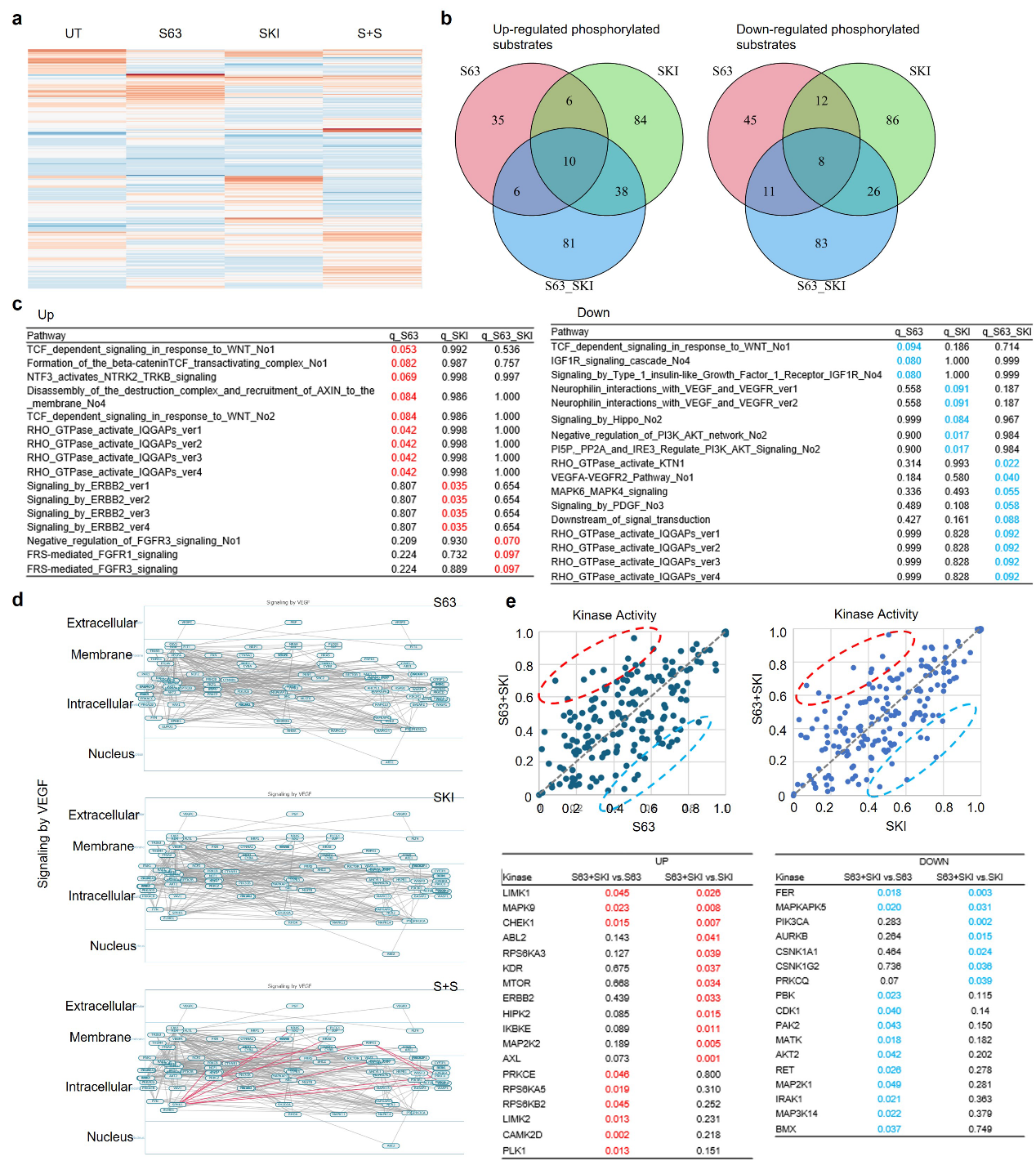
**

**Supplementary Figure S19.**

**Phosphorylation array analysis.**

**a.** Heatmap of phosphorylation states of 1471 substrates across 4 groups.

**b,** Venn diagram illustrates the numbers of differentially phosphorylated substrates (both upregulated and downregulated) across three treatment groups compared to the control group (UT).

**c,** List of significantly up-regulated and down-regulated pathways (*q*<0.05) in the 3 treatment groups compared with the control group (UT).

**d,** A total of 273 pathways were classified into 27 categories, with a focus on the “Signaling by VEGF” pathway, which is visualized here. In the figure, the proteins are arranged from top to bottom in extracellular, membrane, intracellular, and nucleus. Active pathways are indicated by red lines connecting their component proteins, while inactive pathways are shown with gray lines.

**e,** Target kinases with significant activity scores (*p* < 0.05) were identified by comparing the combination treatment to single agents, highlighted in red (increased) and blue (decreased) dashed circles in the figure, and listed in the table.

**Figure S20.**

**
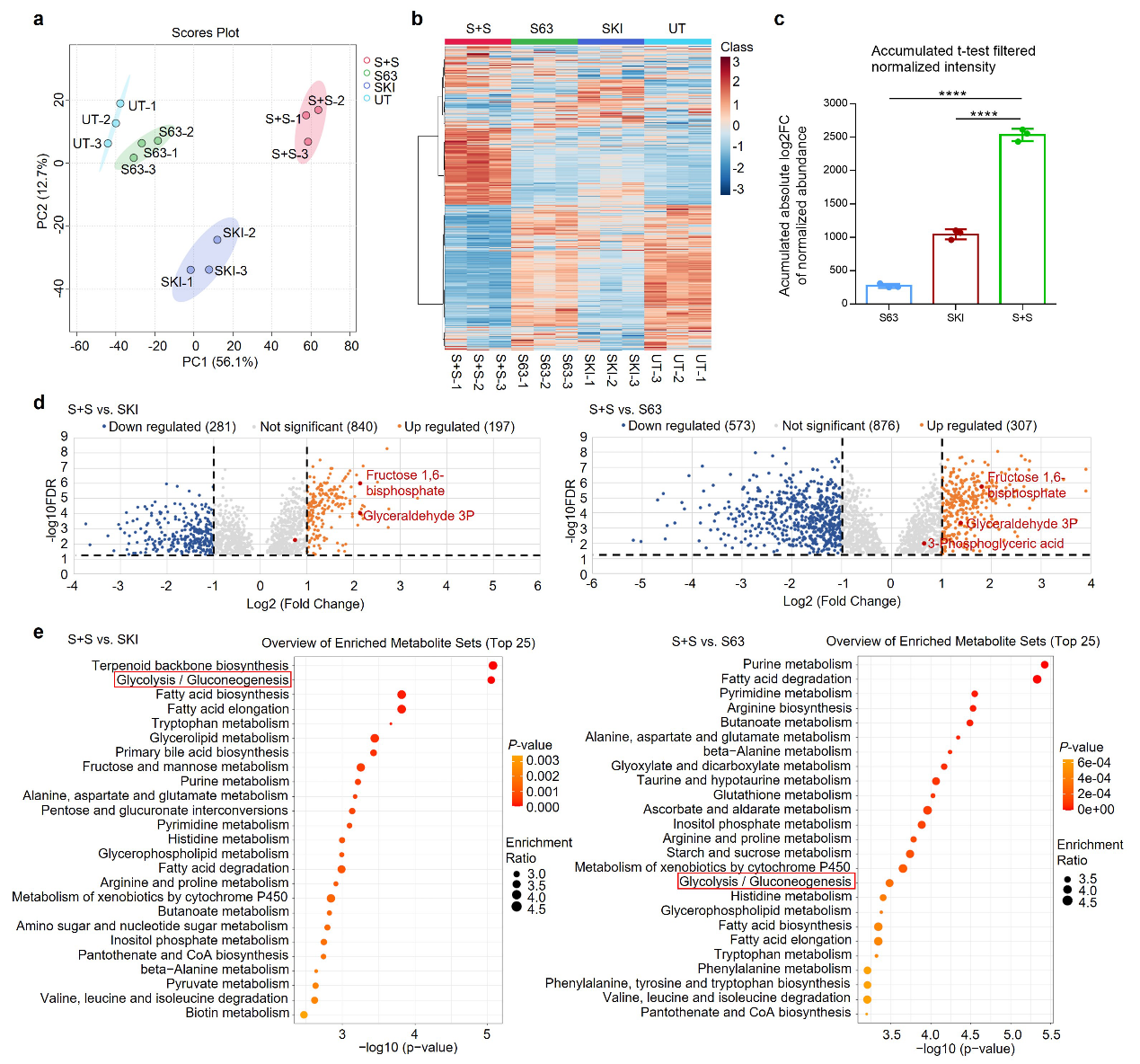
**

**Supplementary Figure S20.**

**Metabolomic profile analysis.**

**a,** PCA two component scores plot of metabolites from control (UT) and treatments (S63, SKI and S+S). The two principal components (PC1 and PC2) represent the most significant variance in the dataset. Each point corresponds to a sample, with distinct colors or shapes indicating the different treatment groups. The clustering of points suggests metabolic differences among the groups, with clear separations indicating significant treatment effects.

**b.** The heatmap illustrates the expression levels of all detected metabolites across the control group (UT) and treatment groups (S63, SKI, and S+S).

**c,** Accumulated t-test filtered log2 fold change(log2FC) of each treatment compared to UT. *****P* <0.0001.

**d,** Volcano plots of S+S vs SKI (left) and S+S vs S63 (right). The x-axis represents the log2 fold change (log2FC), while the y-axis shows the -log10 *p*-value, providing a dual view of effect size and significance. Red represents metabolites that are significantly upregulated; blue represents metabolites that are significantly downregulated, gray indicates the metabolites were not significantly changed.

**e,** Metabolites enrichment analysis of S+S vs SKI and S+S vs S63.
