# Supplementary material for: Src inhibition potentiates MCL-1 antagonist activity in acute myeloid leukemia
Source: Signal Transduct Target Ther. 2025 Feb 10;10:50. doi: 10.1038/s41392-025-02125-x (PMC11808118; doi:10.1038/s41392-025-02125-x)
Supplement: Supplementary file 3 — Supplementary Table S2 [file 41392_2025_2125_MOESM3_ESM.docx]

**Supplementary table S2**

| **Patient #** | **Gender** | **Age** | **Disease** | **Cytogenetics**  **(deletion/translocation)** | **NGS/PCR Result**  **(FLT3, NPM, IDH1/2, CEBPA, RUNX, ASXL1, p53)** | **Prior**  **Treatment** |
| --- | --- | --- | --- | --- | --- | --- |
|  | **(M: 14/32; F: 18/32)** |  | **(new: 15/32; RR: 17/32)** |  |  |  |
| 1 | F | 82 | R/R | N/A | *ASXL1, NRAS, STAG-2* | Ven/Decitabine |
| 2 | M | 31 | new | t(8;21), (q22,q22) | *NPM1-, CEBP1-, FLT3-* | N/A |
| 3 | F | 57 | R/R | trisomy 21, (Inv)3 | *CSF3R, CUX1, SETBP1, STAG3* | 7+3, HIDAC, Ven/Aza, gemtuzumab, Flag/Ida/Ven |
| 4 | F | 36 | R/R | T(8:21) T(5:17) | *RUNX1, ITI, IDH* | 7+3, HIDAC, Flag/IDA, Flag/IDA/Ven, Allo-SCT |
| 5 | M | 70 | new | - | *RUNX1, ABL1* (D872) | N/A |
| 6 | F | 58 | R/R | 5q31, 7q31,20q12,amp 11q23 | *RUNX1, TP53, ETV, NRAS* | 7+3, Aza/Ven, Flag/IDA/Ven |
| 7 | F | 82 | R/R | - | *TP53* | Aza, HU |
| 8 | M | 44 | new | Inv(16) | *CRFB* | N/A |
| 9 | F | 66 | R/R | - | *IDH1* | 7+3, Ven/Aza, Ivosidernib |
| 10 | M | 49 | R/R | - | *IDH2, SR5F2+* | 7+3 |
| 11 | F | 61 | R/R | 11q23, | *KMT2A, FLT3, KRAS/NRAS* | Aza/Ven, Flag/IDA, Cladarabine/ara-C |
| 12 | M | 31 | new | (1)(p12) | - | N/A |
| 13 | M | 61 | new | - | - | N/A |
| 14 | M | 73 | R/R | t(8:21) | *ASXL1, CCND2, NRAS* | Aza/VEN |
| 15 | M | 53 | new | t(6;9) | *FLT-ITD* | N/A |
| 16 | F | 76 | R/R | - | *RUNX1* | Ven/Aza, decitabine/Ven |
| 17 | F | 70 | R/R | - | - | Aza/Ven |
| 18 | M | 47 | new | - | - | N/A |
| 19 | F | 82 | new | - | - | N/A |
| 20 | F | 36 | new | - | - | N/A |
| 21 | F | 47 | R/R | - | - | 7+3 |
| 22 | M | 60 | R/R | - | *FLT3* | 7+3 +mid, Aza/Ven, Flag/IDA/Ven, gliteritinib |
| 23 | F | 81 | R/R | - | *FLT3, NPM1* | decitabine, Ven/Aza |
| 24 | F | 70 | new | monosomy 7 | - | N/A |
| 25 | F | 78 | R/R | complex karyotype | *TP53* | decitabine/Ven |
| 26 | F | 57 | R/R | trisomy 21 | *CSF3R* | 7+3, Ven/Aza, HIDAC, gemtuzumab, Flag/Ida/Ven |
| 27 | M | 67 | New | gain of signal MYC | - | N/A |
| 28 | M | 55 | R/R | - | *FLT3, NPM1, DNMT3A* | 7+3, stem cell transplantation |
| 29 | F | 79 | New | - | - | N/A |
| 30 | M | 70 | new | trisomy 21; monosomy 18;17p13 (TP53) deletion [67%] | *TP53 R175H* | N/A |
| 31 | M | 71 | new | trisomy 14 | *NRAS G13R* | N/A |
| 32 | F | 66 | new | - | *FLT3, IDH1, NPM1* | N/A |
| RR: relapsed or refractory; N/A: not applicable | | | | | | |

**Supplemental Table S2.** Clinical, molecular, and cytogenetic characteristics of patient samples exposed to MCL-1 inhibitors ± SKI-606.
