# Supplementary material for: Src inhibition potentiates MCL-1 antagonist activity in acute myeloid leukemia
Source: Signal Transduct Target Ther. 2025 Feb 10;10:50. doi: 10.1038/s41392-025-02125-x (PMC11808118; doi:10.1038/s41392-025-02125-x)
Supplement: Supplementary file 4 — Supplementary Table S3 [file 41392_2025_2125_MOESM4_ESM.docx]

**Supplementary table S3**

| **Patient #** | **Gender** | **Age** | **Disease** | **Cytogenetics**  **(deletion/translocation)** | **NGS/PCR Result**  **(FLT3, NPM, IDH1/2, CEBPA, RUNX, ASXL1, p53)** | **Prior**  **Treatment** |
| --- | --- | --- | --- | --- | --- | --- |
| 1 | M | 77 | new | t(11;19) | *MLL (KMT2)* | N/A |
| 2 | M | 55 | R/R | - | *NPM1, FLT3* | Ara-C, DNR, midostaurin |
| 3 | F | 81 | new | - | *-* | N/A |
| RR: relapsed or refractory; N/A: not applicable | | | | | | |

**Supplemental Table S3.** Clinical, molecular, and cytogenetic characteristics of patient samples used for PDX models.
