# Supplementary material for: Src inhibition potentiates MCL-1 antagonist activity in acute myeloid leukemia
Source: Signal Transduct Target Ther. 2025 Feb 10;10:50. doi: 10.1038/s41392-025-02125-x (PMC11808118; doi:10.1038/s41392-025-02125-x)
Supplement: Supplementary file 5 — Supplementary Table S4 [file 41392_2025_2125_MOESM5_ESM.docx]

**Supplementary Table S4**

| **Pathway** | **Category** | ***p*_S63** | | ***p*_SKI** | ***p*_S63_SKI** | | |
| --- | --- | --- | --- | --- | --- | --- | --- |
| NRAGE_signals_death_through_JNK_No1 | Deth Receptor Signaling | 0.68 | | 0.1 | 0.1 | | |
| GRB2_SOS_provides_linkage_to_MAPK_signaling_for_Integrins_ver1 | Integrin signaling | 0.98 | | 0.01 | 0.03 | | |
| CaMK_IV-mediated_phosphorylation_of_CREB_No2 | Intracellular signaling by second messengers | 0.25 | | 0.29 | 0.13 | | |
| Cam-PDE1_activation | Intracellular signaling by second messengers | 0.25 | | 0.36 | 0.01 | | |
| Negative_regulation_of_MAPK_pathway_No3 | MAPK Family signaling Cascades | 0.92 | | 0.79 | 0.01 | | |
| IRS_Activation | Signaling by Insulin receptor | 0.88 | | 0.02 | 0.13 | | |
| PTK6_Actives_STAT3 | Signaling by Non-Receptor Tyrosine Kinase | 0.52 | | 0.81 | 0.12 | | |
| PTK6_Regulates_Cell_Cycle | Signaling by Non-Receptor Tyrosine Kinase | 0.8 | | 0.64 | 0.17 | | |
| PTK6_Regulates_RHO_GTPases,_RAS_GTPase_and_MAP_kinases | Signaling by Non-Receptor Tyrosine Kinase | 0.01 | | 0.5 | 0.01 | | |
| Downstream_of_signal_transduction | Signaling by PDGF | 0.05 | | 0.01 | 0.01 | | |
| Signaling_by_PDGF_No3 | Signaling by PDGF | 0.09 | | 0.09 | 0.02 | | |
| VEGFA-VEGFR2_Pathway_No5 | Signaling by VEGF | 0.67 | | 0.43 | 0.01 | | |
| VEGFR2_mediated_cell_proliferation_No2 | Signaling by VEGF | 0.65 | | 0.38 | 0.03 | | |
| Binding_of_TCF_LEF_CTNNB1_to_target_gene_promoters_No1 | Signaling by WNT | 0.15 | | 0.82 | 0.16 | | |
| Negative_regulation_of_TCF-dependent_signaling_by_DVL-interacting_proteins_ver1 | Signaling by WNT | 0.79 | | 0.5 | 0.12 | | |
| FasL_CD95L_signaling | Deth Receptor Signaling | | 0.68 | 0.01 | | 0.84 |  |
| Signaing_by_Leptin | Signaing by Leptin | | 0.37 | 0.08 | | 0.28 |  |
| GAB1_signalosome | Signaling by EGFR | | 1 | 0.62 | | 0.78 |  |
| MET_activates_PTPN11_ver1 | Signaling by MET | | 0.96 | 0.99 | | 1 |  |
| ERK_MAPK_targets_No2 | Signaling by NTRKs | | 0.01 | 0.52 | | 1 |  |
| Disassembly_of_the_destruction_complex_and_recruitment_of_AXIN_to_the_membrane_No2 | Signaling by WNT | | 1 | 0.1 | | 0.99 |  |
| Disassembly_of_the_destruction_complex_and_recruitment_of_AXIN_to_the_membrane_No5 | Signaling by WNT | | 0.98 | 0.01 | | 0.38 |  |
| TCF_dependent_signaling_in_response_to_WNT_No2 | Signaling by WNT | | 1 | 0.17 | | 0.97 |  |
| TCF_dependent_signaling_in_response_to_WNT_No5 | Signaling by WNT | | 0.97 | 0.02 | | 0.3 |  |
| RHO_GTPase_activate_CFTRtrafficking | Signalingby Rho GTPases | | 0.01 | 0.01 | | 0.63 |  |
| RHO_GTPase_activate_IQGAPs_ver2 | Signalingby Rho GTPases | | 1 | 0.19 | | 0.93 |  |
| RHO_GTPase_activate_IQGAPs_ver3 | Signalingby Rho GTPases | | 1 | 0.17 | | 0.95 |  |

**Supplemental Table S4.** List of active and inactive pathways of (S63+SKI) group compared with those of S63 and SKI groups.
